# Crosslinking, salt-induced aging, and secondary structure formation in Peptide-containing coacervates inspired by spider silk

**DOI:** 10.1038/s42004-025-01634-8

**Published:** 2025-08-28

**Authors:** Armin Amirsadeghi, Raffaella Parlato, Anna Kenbeek, Ana Rita Gaspar, Marta Oggioni, Alessia Lasorsa, Adrivit Mukherjee, Malak Jaber, Małgorzata K. Włodarczyk-Biegun, Patrick C. A. van der Wel, Marleen Kamperman, Guillermo Monreal Santiago

**Affiliations:** 1 Polymer Science, Zernike Institute for Advanced Materials, University of Groningen, Groningen, The Netherlands; 2 Solid-state Nuclear Magnetic Resonance, Zernike Institute for Advanced Materials, University of Groningen, Groningen, The Netherlands; 3 UMR7140 - Chimie de la Matière Complexe, CNRS, Université de Strasbourg, Strasbourg, France; 4 Biotechnology Centre, The Silesian University of Technology, Gliwice, Poland

**Keywords:** Polymer characterization, Rheology, Polymers, Mechanical properties

## Abstract

Spider silks are exceptional biomaterials: biocompatible, biodegradable, and with remarkable mechanical properties. Unfortunately, attempts to replicate them tend to fail due to the difficulty of synthesizing the proteins that constitute them, and to an incomplete understanding of their processing conditions. Here, we report a synthetic system inspired by spider silk, consisting of a synthetic polyelectrolyte with grafted oligoalanine chains. We have used this peptide-polyelectrolyte conjugate to produce complex coacervates in an analogous process to the liquid-liquid phase separation (LLPS) observed during the natural processing of spider silk. We have characterized these coacervates using rheology, tack test, and solid-state NMR spectroscopy, observing α-helixes and *β*-sheets. These secondary structures crosslink the material, improving its mechanical properties and its processability, for example, for 3D printing. Furthermore, the peptide-based crosslinks cause distinctive behaviours – such as salt-induced aging. Our approach contributes to the fundamental understanding of the role that LLPS and peptide crosslinks play in spider silk, and to the development of new soft materials crosslinked by peptide aggregation.

## Abstract (web summary)

Spider silks are attractive candidates as biomaterials due to their biocompatibility, biodegradability and mechanical properties, but synthesizing their protein constituents remains challenging. Here, the authors develop a minimal synthetic mimic that undergoes complex coacervation and supramolecular crosslinking, revealing spider silk-like behaviors such as liquid–liquid phase separation, secondary structure formation, and aging.

## Introduction

Spider silks have high tensile strength and stretchability, comparable with the strongest synthetic fibers^1,2^, and are biocompatible and biodegradable^3,4^. The combination of these features makes spider silk suitable for various biomedical applications such as nerve^5^, skin^6^, or bone regeneration^7^. However, producing spider silk in large amounts is a challenging process. Harvesting it is unfeasible due to its low production rate and the territorial and cannibalistic behavior of spiders^8^. The recombinant production of spidroins (the proteins that compose spider silk) is the most commonly used alternative^9–11^, but it also suffers from a few shortcomings. First, the composition of spidroins is unusual, as over 60% of the residues are alanine and glycine. This limits their expression in most cells, which do not have a high enough stock of the corresponding tRNAs^12^. Second, spidroins are remarkably long and contain hydrophobic patches (see below), which leads to low solubility. Inside spiders, the proteins are stored in specialized glands, where they remain soluble until they are processed into the final material. The process through which the proteins are stored and processed while avoiding precipitation has not yet been completely understood or replicated in a synthetic system^2,13,14^.

**Fig. 1:**
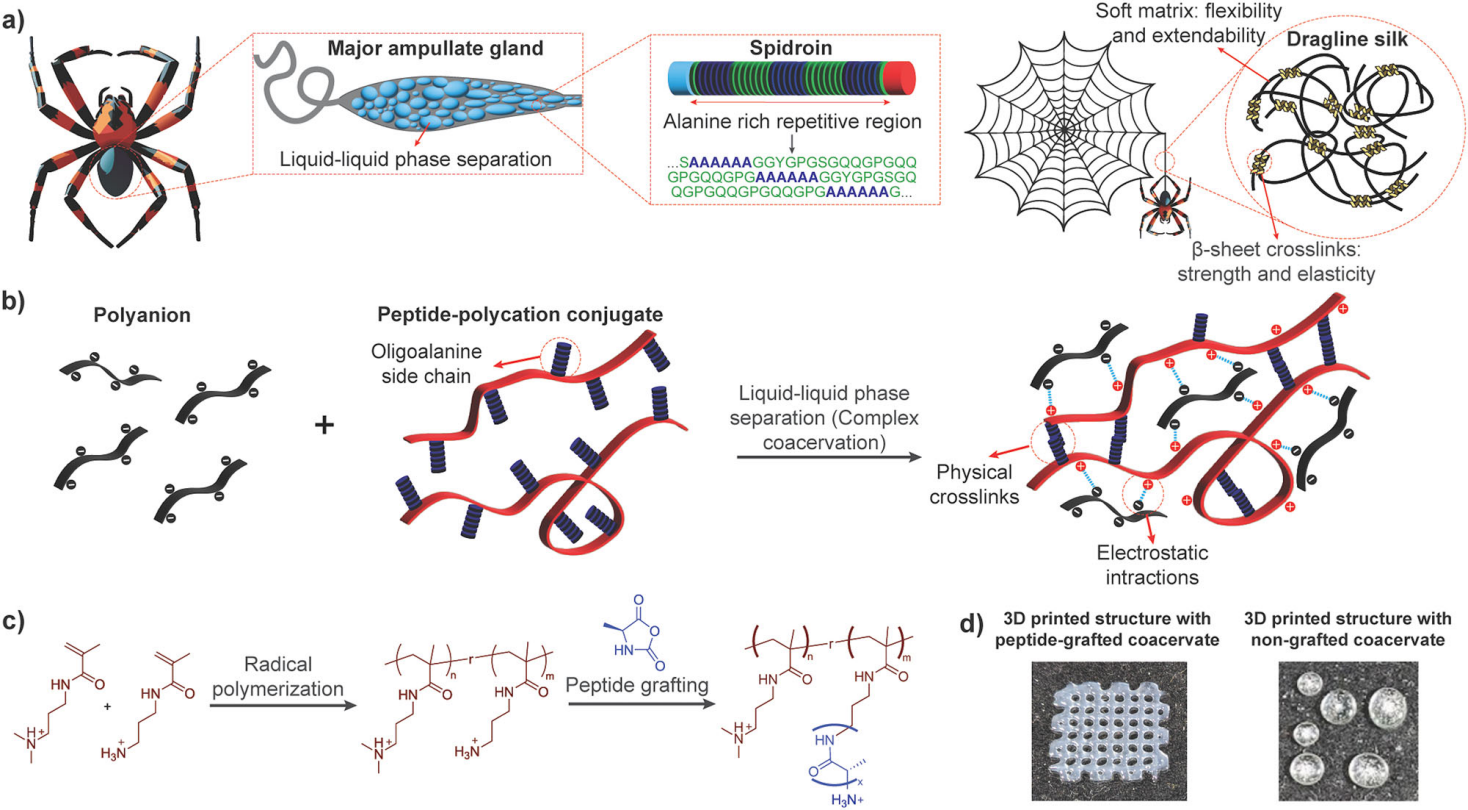
Synthesis, structure, and processability of peptide-containing coacervates inspired by spider silk. **a** Schematic representation of some of the key elements in spider silk production: the liquid-liquid phase separation of spidroins inside of the major ampullate gland leads to a liquid, highly-concentrated spinning dope. This spinning dope contains only spidroins, proteins with a repeating structure that alternates oligoalanines with hydrophilic sequences. In the final material, the hydrophilic sequences form a soft matrix, while the oligoalanines form *β*-sheet crystals that crosslink the material. **b** Bioinspired design of peptide-containing complex coacervates. The polymers synthesized in this work combine a polyelectrolyte backbone with grafted oligoalanine chains. The polyelectrolyte backbone allows for complex coacervation, a type of LLPS analogous to the phase separation observed in spider silk. The oligoalanines lead to *β*-sheet formation and crosslinking of the material. **c** Scheme of the reactions leading to the synthesis of the final material. **d** 3D printed structures of coacervates with and without grafted peptides, 15 min after printing. As it can be observed, only the peptide-containing coacervates were able to retain any structural definition, while the coacervates without peptides relaxed quickly into droplets.

Both the chemical composition and the processing of spider silk are necessary to achieve its unique material properties^14–17^. Regarding structure, dragline silk (the most commonly studied spider silk, with a structural function)^17^ contains only spidroins with a molecular weight of 250–350 kDa. The primary structure of these spidroins consists of two short nonrepetitive terminal domains and one long repetitive domain, which alternates oligoalanine blocks with blocks rich in glycine (Fig. 1a)^13,17^. The hydrophobic oligoalanine blocks can form *β*-sheet crystals containing different spidroins, crosslinking the final material and increasing its strength, while the hydrophilic glycine-rich blocks make the silk flexible and extensible. Some efforts have been made to incorporate these structural elements into synthetic materials to improve their mechanical properties. For example, Chan et al. grafted *β*-sheet-forming peptides on an already crosslinked hydrogel, observing a significant improvement in its compressive modulus^18^. Gu et al. also showed a significant enhancement in the mechanical properties of polyurethane fibers when introducing *β*-sheet-forming peptides^19^.

In terms of processing, the spinning of spider silk takes place in different steps. First, spidroins are synthesized and stored inside specialized glands, forming a viscous spinning dope that contains high protein concentrations. Despite the low solubility of spidroins, the spinning dope remains liquid, avoiding aggregation and precipitation. The protein at this stage does not contain *β*-sheet crosslinks, which are formed through the spinning process due to a combination of salt concentration changes, pH change, shear force, and slow conformational changes known as aging^20,21^. Despite multiple efforts, the exact mechanism for the formation of this spinning dope has not been elucidated or replicated in the laboratory, with synthetic protocols often using organic solvents to prepare concentrated spidroin solutions^22^. Recent studies have made substantial progress towards replicating the synthesis and processing of natural spider silk^23–25^. While natural proteins face problems during expression due to their unusually large length, smaller recombinant versions bearing both repetitive domains and globular terminal domains have been expressed at high yields, and assembled into fibers entirely under aqueous conditions^26–29^. In order to replicate the natural processing conditions, natural and synthetic spidroins have been subjected to the same sequence of chemical triggers that take place within the spider’s gland: addition of kosmotropic ions, changes in pH and shear-induced β-sheet formation^30–32^. These biomimetic approaches have enabled the production of highly extensible artificial silk fibers with toughness values approaching or even surpassing those of native dragline silk^29^. Liquid-liquid phase separation (LLPS) plays a key role in both natural and artificial processing of spidroins^26,31,33^. Through LLPS, a concentrated solution of protein spontaneously decomposes into two inmiscible aqueous phases. This process has been observed for multiple proteins^34^, particularly those containing polyalanine repeats^35,36^.

There are different processes that can lead to LLPS, based on either associative or segregative interactions. In spider silk, LLPS takes place through a combination of salting-out effects and weak interactions between “sticker” sequences that are separated from each other^31^. The process is complex, and different mechanisms have been proposed, including the formation of a liquid-crystalline phase^37,38^. In synthetic materials, LLPS is commonly achieved through complex coacervation^39,40^. In complex coacervation, the associative electrostatic interactions between two oppositely charged polyelectrolytes drive the formation of two water-based phases: one concentrated in polyelectrolytes (also known as dense phase, or coacervate), and a diluted one (also called supernatant). This type of LLPS has been observed in multiple animals that excrete biomaterials, such as mussels^41,42^ and sandcastle worms^43^. Complex coacervation has also been used as a green and mild approach toward the production of synthetic materials, as it spontaneously leads to the formation of a polymer dope with high concentration, only requires water as a solvent and salt as a plasticizer, and takes place without any heating^44^. We have recently shown that complex coacervation can be used to process natural proteins into fibers^45^ and to make 3D-bioprinted scaffolds for biomedical applications^46^.

The complexity of the systems containing spider silk moieties makes them highly challenging to obtain information such as composition, secondary structure, mobility, etc. The conformational and dynamic disorder of these samples prevents the determination of atomic-resolution information by X-ray crystallography or electron microscopy. In such conditions, spectroscopic techniques can be deployed to probe the polypeptide secondary structures, such as *β*-sheets, *α*-helixes, and random coil structures, which are expected in spider silk^47^. Vibrational spectroscopies, such as infrared spectroscopy, permit the detection of secondary structural motifs, but peak overlap limits their use on these chemically and structurally complex samples. Moreover, these techniques would not provide insights into the dynamics and interactions that underpin the expected roles of the silk-inspired peptides. Instead, here we employed magic-angle-spinning (MAS) solid-state NMR (ssNMR), which has been extensively used to analyze the molecular and structural aspects of diverse types of silk-based polypeptides^48,49^. Using MAS ssNMR, it is possible to dissect secondary structure, molecular mobility, composition, and interactions of peptides with atomic-level resolution. Moreover, selective isotope labeling (with stable isotopes ^13^C and ^15^N) of the peptide component of complex hybrid materials permits the targeted analysis of their molecular features with minimal interference from the (unlabeled) polymer matrix.

In this work, we have synthesized a simple system inspired by spider silk, which combines some of its structural elements with the ability to phase separate through complex coacervation (Fig. 1b). In particular, our design is based on a peptide-polyelectrolyte conjugate, which combines a polycation backbone with grafted oligoalanine chains. The positively charged backbone can adopt a random chain configuration, playing a similar role to the flexible regions in spidroins, while the oligoalanines can crosslink the material through the formation of *β*-sheets and other secondary structures. LLPS can be induced in this system by the addition of a polyanion, leading to the formation of a complex coacervate, which can then be further processed into fibers or 3D-printed structures. By combining insights from rheology and ssNMR, we have established the mechanism through which crosslinking and aging occur in these materials.

Our goal is not to create a material with mechanical properties equivalent to natural spider silk, but to develop a minimal system that we could use to understand how the combination of LLPS and crosslinking between oligopeptides leads to enhanced mechanical properties. The system developed here shows how the introduction of oligoalanines in a coacervate can lead to crosslinking and salt-induced solidification. Furthermore, the synthetic strategy used here can be used to introduce oligoalanine crosslinks in other materials with improved mechanical properties. Finally, studying how different elements (e.g., shear, alignment) affect the mechanical properties of our materials can provide us with new insights on the processing of natural silk.

## Results

### Synthesis of a peptide-containing polyelectrolyte inspired by spider silk

The composition of spidroins varies between different species of spiders, and between silks with different functions. However, structural silks normally contain blocks of oligoalanines, alternating with blocks of hydrophilic amino acids. In order to imitate this structure, we designed complex coacervates where the polyelectrolytes contain grafted oligoalanine chains of a certain length. For the sake of simplicity and to allow for more mobility in our materials, we only grafted alanines onto one of the polyelectrolytes, in this case the polycation. This was achieved in a two-step process: first copolymerization and then grafting using N-carboxyanhydride (NCA) ring-opening polymerization (Fig. 1c and Scheme S1). NCA polymerization is a widely utilized method for synthesizing polypeptides and polymer-peptide conjugates, offering precise control over chain length, composition, and architecture^50^. This reaction is initiated by a nucleophile, such as a primary amine, onto which the peptide chain is grafted. In our case, we introduced these initiators randomly in the backbone chain, using free radical polymerization. The advantage of this strategy is that it allowed us to control both the number of grafting points in the backbone (by adjusting the ratio between primary amine and tertiary amine monomers) and the average length of the oligoalanine chains at each grafting point (by adjusting the ratio between grafting points and Ala-NCA). An initial screening indeed showed a linear relationship between the monomer ratio and the number of grafting points incorporated into the backbone (up to at least 19%, see Fig. S2), as well as between the concentration of Ala-NCA and the alanine: backbone ratio in the final sample (see Fig. S4). The possibility of easily controlling the density of grafted chains and their length opens the door for further tuning the mechanical properties of these materials with minimal synthetic effort.

For this work, we centered our studies on a polymer with a targeted percentage of grafting points of 5%, and a targeted oligoalanine length of 6, to which we will refer further as grafted polymer backbone, or GPB. These numbers were selected because they correspond roughly to the alanine content of eADF3, the main structural spidroin for the spider Araneus diadematus^51^. The sequence of this protein contains a hexaalanine block every 40 amino acids, while in our case a grafting percentage of 5% would achieve 6 alanines per every 40 charges after complexation.

The synthesized polymers were characterized by ^1^H-NMR and DOSY-NMR, observing that while the ratio between alanine residues and grafting points was similar to the expected one (Fig. S4), roughly 25% of the alanines were not grafted to the backbone (Fig. S5). This is a consequence of the self-initiation of Ala-NCA polymerization in presence of bases, and cannot be avoided^50^.

For most experiments, GPB was not purified further and contained these non-grafted oligoalanine chains. However, in order to determine the different effects of grafted and non-grafted oligoalanine chains on the mechanical properties, we also prepared two controls: one containing only grafted oligoalanines (purified GPB, or P-GPB) and another one containing only non-grafted chains (referred to as homopolymer + non-grafted alanines, HP + NG-Ala).

The synthesized systems are evidently different than spidroins – the oligoalanines are grafted as side-chains rather than incorporated in the background, and they are randomly spaced rather than in regular intervals. However, this is compensated with the simplicity and the versatility of the synthesis, which allows us to prepare grams of material in only two steps, or to modify the polymer architecture in the ways described above.

### Complex coacervation of the peptide-polyelectrolyte conjugates

The effect of the oligoalanine chains on the properties of the polymer was already apparent before LLPS. Both the homopolymer containing only charged units (HP), and the polymer backbone before peptide grafting (PB) were completely soluble in water independent of the pH. However, GPB was completely insoluble at high pHs (≥9), and could only be redispersed into a homogeneous dispersion in neutral or acidic conditions. In order to imitate the phase separation of spidroins and their formation of a viscous dope, we induced complex coacervation in our system by the addition of a polyanion, polyacrylic acid (pAA). Despite the low solubility of GPB, mixing both polyelectrolytes resulted in a dispersion of coacervates that could be centrifuged into a homogeneous phase. A characteristic parameter of complex coacervates is their salt resistance: the salt concentration above which they dissolve, due to the screening of electrostatic interactions. Similar coacervates prepared by our group^52^ have shown salt resistances of ca. 0.7 M. This was also the case for the coacervates formed using HP (Fig. S7). In the case of GPB/pAA, the coacervates were not completely solubilized even at high salt concentrations (above 3 M). However, their appearance changed drastically when the salt concentration increased further than 0.7 M, forming a fine dispersion rather than a coalesced phase (Fig. S8). Our explanation for this lack of a coacervate-to-solution transition in GPB/pAA coacervates is that, even though high salt concentrations are able to screen the charges and disrupt the electrostatic interactions in this system, the oligoalanine chains that are grafted to the polymer chains remain attached to each other, forming secondary structures and causing aggregation. Interestingly, the fact that the electrostatic interactions break at the same salt concentration in both systems highlights that the two types of interactions (peptide crosslinks and electrostatic crosslinks) are completely orthogonal to each other, and can be influenced independently.

### Peptide crosslinking causes an increase in mechanical properties and salt-dependent aging

**Fig. 2:**
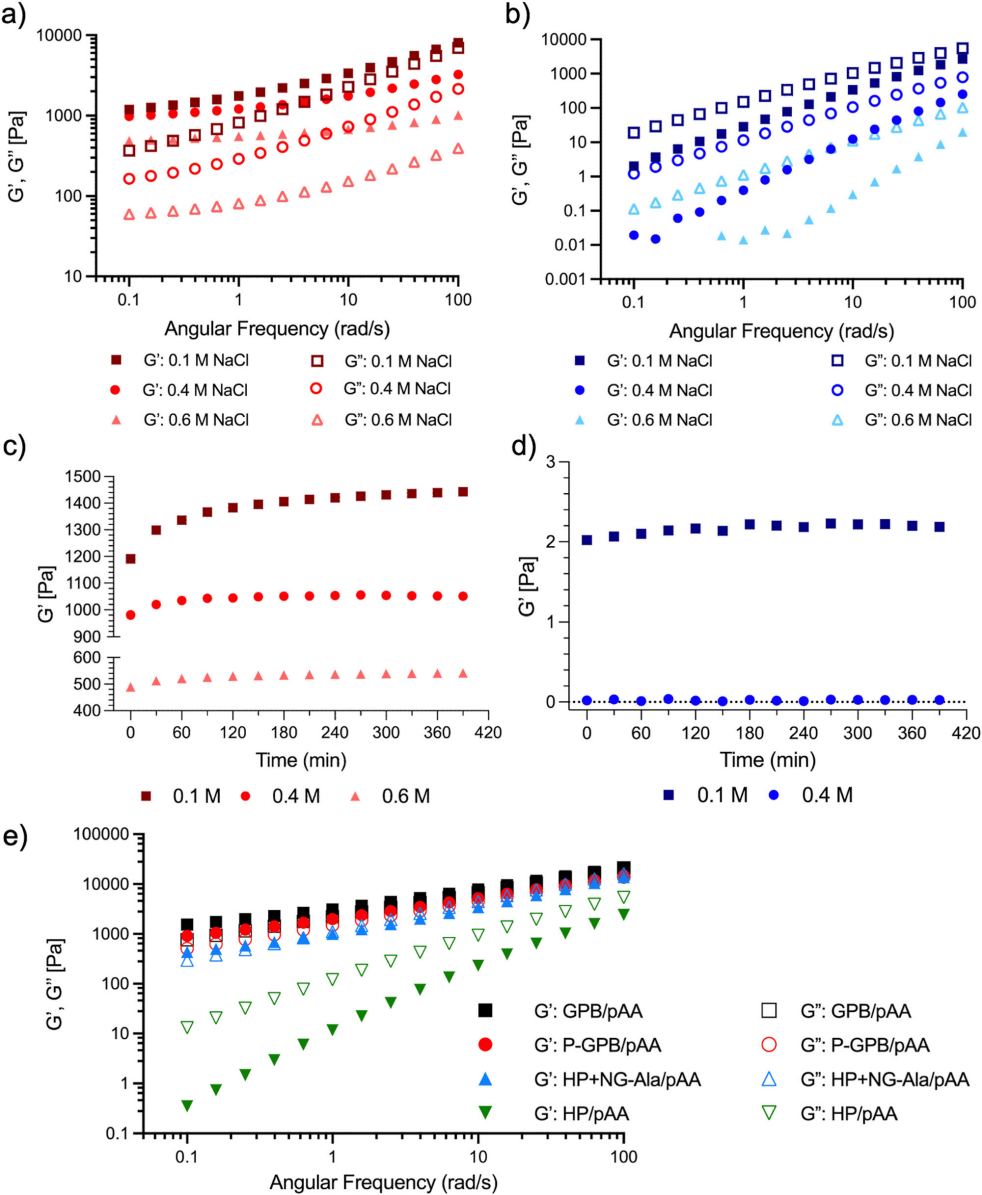
Oligoalanine chains cause crosslinking and salt-dependent aging. Frequency sweep measurements of **a** GPB/pAA and **b** HP/pAA coacervates at 0.1, 0.4, and 0.6 M NaCl. **c** Temporal evolution of the storage modulus (G’) of GPB/pAA coacervates at different salt concentrations. **d** Temporal evolution of the storage modulus (G’) of HP/pAA coacervates at different salt concentrations. **e** Frequency sweep measurements of GPB/pAA, P-GPB/pAA, HP + NG-Ala/pAA, and HP/pAA, at a NaCl concentration of 0.1 M. The filled symbols representing the storage modulus (G’) and hollow symbols representing the loss modulus (G”). The pH was adjusted to 7 for all coacervates. All the measurements were performed with a constant strain of 1%, within the linear viscoelastic regime.

To study the mechanical properties of the prepared coacervates, we centrifuged them to obtain a separate phase and studied them using oscillatory rheology. The dynamic frequency sweep measurements of different coacervates are shown in Fig. 2. As expected, the HP/pAA coacervates (Fig. 2b) behaved as viscoelastic liquids, showing low moduli and G” > G’ across the entire frequency range. Increasing the salt concentration in these coacervates weakened them, reducing their moduli further. However, grafting peptide chains in these coacervates improved their mechanical properties drastically (Fig. 2a). First, the moduli of all GPB/pAA coacervates increased by three orders of magnitude compared to their counterparts without peptide. Secondly, all of these materials behaved like viscoelastic solids for the entire range of frequencies explored (G’ > G”). Additionally, the peptide-containing coacervates were more resistant to salt: even though a decrease in G’ was observed when the salt concentration increased, all samples remained as viscoelastic solids even at 0.6 M NaCl (close to the disruption of electrostatic interactions).

In order to determine the contribution of the non-grafted oligoalanines contained in GPB toward the observed improvement in mechanical properties, we also performed the same measurement on samples containing either only grafted oligoalanines (P-GPB/pAA), or only non-grafted oligoalanines (HP + NG-Ala/pAA) (Fig. 2e). We found that the moduli of both of these controls were lower than GPB/pAA, indicating that grafted and ungrafted oligoalanines have a synergistic effect toward crosslinking the material. Our hypothesis is that, in this case, the non-grafted peptides participate in the formation of larger secondary structures, which include peptides from different polymer chains and contribute towards their crosslinking. Surprisingly, the coacervate with only non-grafted peptides (HP + NG-Ala/pAA), even though it was the weakest of the three, had a modulus comparable to the other two and still behaved like a viscoelastic solid. Our explanation for this is that the non-grafted chains can form aggregates by themselves, which are then embedded in the polymer matrix, reinforcing the material as a “physical” crosslinker. Overall, these results not only show the effect of peptide crosslinkers in the mechanical properties of complex coacervates but also highlight the importance of how exactly the peptides are incorporated into the coacervate structure.

Since all peptide-containing coacervates behaved like viscoelastic solids at rest, they were suitable for 3D printing^46^. To test the printability of these materials, we prepared both GPB/pAA and HP/pAA coacervates and attempted to use them to 3D-print a square grid (Fig. 1d). Both coacervates were printed on a Petri dish using a conventional extrusion bioprinter, and dried in air. As it can be observed in that Figure, the coacervates without peptides relaxed very quickly, losing the definition of the originally printed structure. On the other hand, the coacervates formed from GPB and pAA remained stable, keeping the original design.

Peptide-based condensates typically exist in a metastable state, in which the material slowly self-assembles after phase separation, forming a more thermodynamically stable structure. This process, often referred to as aging, gradually alters the mechanical properties of the material^53^. We hypothesized that similar aging process could be taking place in our system, as the polymeric matrix might need time to rearrange itself to form the most stable form of intermolecular crosslinks. To investigate this possibility, we performed a time-dependent series of frequency sweep experiments on GPB/pAA and HP/pAA coacervates (Figs. 2c, d, and S9). Our results show that aging does indeed occur in the peptide-containing coacervates, which become more solid over the first hours of the experiment. This time-dependent evolution of mechanical properties is indicative of a metastable state, where the material gradually evolves through internal structural rearrangements. This metastability results from the fact that there are two types of interactions: electrostatic and peptide-peptide aggregation. Immediately after mixing, the coacervates are mainly held together by electrostatics, but over time, they evolve slowly towards a more thermodynamically stable state in which the peptides are also aggregated. Interestingly, the time that was necessary to reach equilibrium depended on the salt concentration. At lower salt concentrations (0.1 M), reaching equilibrium took up to 6 hours, while samples containing more salt (0.4 M) equilibrated in less than 1 h. This results in a counterintuitive observation for a complex coacervate: increasing the salt concentration leads to faster solidification. The salt-dependency of this aging process can be explained by the plasticizing effect of salts^54^. Decreasing salt concentration in coacervates strengthens the electrostatic interactions between polyelectrolytes and thus makes the coacervates more solid-like. This slows down molecular movement and, in our case, increases the time necessary for the peptides to find each other in the right conformation to crosslink the material. Moreover, peptide-free coacervates of similar composition did not exhibit any change over time, confirming that the metastability is introduced specifically by peptide-mediated interactions and structural transitions (Fig. 2d and Fig. S9d, e).

Aging has been reported for both natural^55^ and recombinant silk proteins^21^. In this case, aging is a consequence of both metastability and chemical changes (e.g., degradation)^55^. Metastability has also been described in synthetic coacervates, especially those containing polyelectrolytes with high molecular weight or strongly interacting motifs such as peptides^56–58^. However, the aging mechanism of the current system differs from these examples as it is based on two different supramolecular interactions (electrostatic and peptide-peptide aggregation) which take place over very different time scales. Since one of which can be modulated but not the other, we can control both the kinetics of aging and the final properties of the material. This opens new possibilities for silk-inspired processing, where, for example, the material becomes stronger once it has been spun into fibers.

### Deformation outside of the linear viscoelasticity regime

**Fig. 3:**
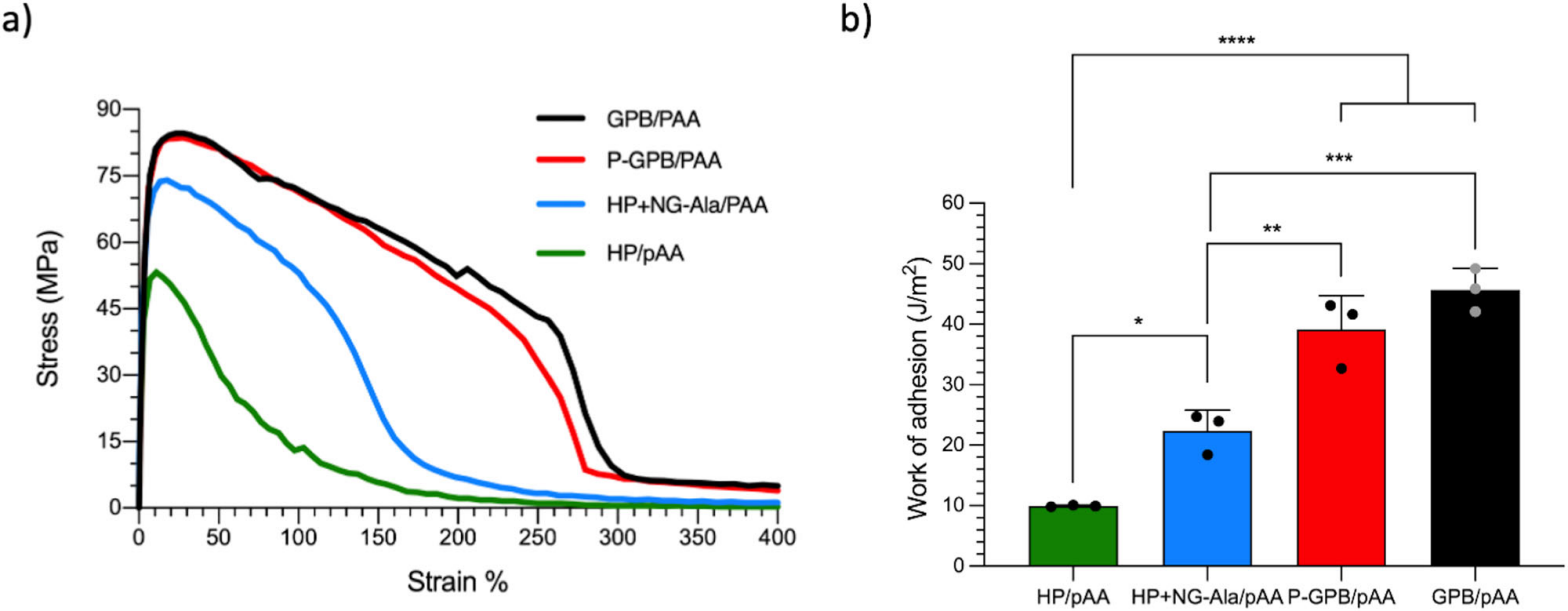
Peptide chains stabilize coacervates towards deformations outside of the linear viscoelasticity regime. **a** Stress-strain curve obtained from a tack experiment of different coacervates. **b** Work of adhesion, obtained from calculating the area under the curve from the stress-strain curve. The data reported for the work of adhesion is the average of three independent measurements, and the error bar corresponds to the standard deviation. Individual data points are shown as dots.

A fundamental factor of the processing of spidroins into silk fibers is the shear forces that are generated during their extrusion, which deform the material outside of the linear deformation regime and induce alignment and secondary structure formation. In order to study the mechanical behavior at high shear of the coacervates prepared above, we studied them using probe tack test (Fig. 3). This test measures the work that is necessary to deform a layer of a sticky substance until the point where it breaks. It is regularly used to determine the strength of adhesives, but it also provides pivotal information about the strength of samples under large-scale deformations. The results of this test showed that oligoalanines (whether grafted or non-grafted) also improve the mechanical properties of the samples under large-scale deformations. More specifically, the addition of oligoalanines improves both the maximum force at break (Fig. 3a) and the work of adhesion (Fig. 3b). The tack test results are in agreement with the results obtained from rheology, confirming the formation of peptide-based crosslinks, and subsequent improvement of mechanical properties.

Interestingly, in this case, the difference between grafted oligoalanines (in GPB/pAA or p-GPB/pAA) and non-grafted oligoalanines (in HP + NG-Ala/pAA) became much more pronounced. We hypothesize that the difference between grafted and non-grafted peptides is related to the crosslinking mechanisms of both materials. In both cases, we expect the oligoalanines to form large aggregates (e.g., *β*-sheets), which are tightly bound and strong. In the case of spider silk, these elements form crystal-like structures that incorporate the main chain of the protein, efficiently crosslinking it^59^. In the grafted systems, the same type of crosslinking can take place, where the polymer fibers are covalently attached to peptides forming secondary and quaternary (i.e., supramolecular) structures, making the coacervates resistant to also strong shear forces. On the other hand, while the peptides in HP + NA-Ala/pAA can still self-assemble and form supramolecular structures, those are only embedded in the polymeric matrix, but not covalently linked to it. While this type of physical entanglement is enough to strengthen the material at low shears (within the linear viscoelasticity regime), it fails to do so at higher deformations. This hypothesis is confirmed by the fact that, in the tack test experiments, the curves corresponding to GPB/pAA and p-GPB/pAA are practically identical – showing that the difference between them (non-grafted oligoalanines) does not have a significative effect.

### Secondary structure of the oligoalanine peptides

**Fig. 4:**
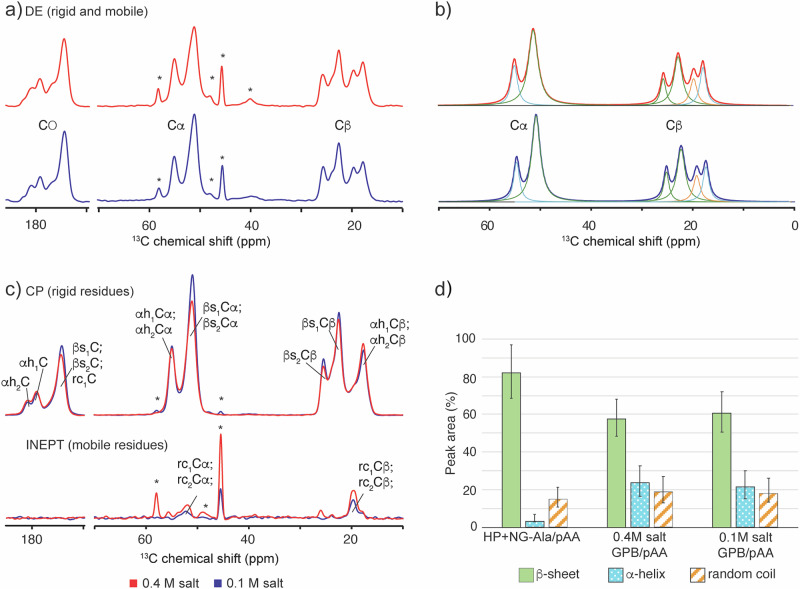
The oligoalanine chains in complex coacervates form rigid aggregates with different secondary structures. 1D ^13^C MAS ssNMR experiments and secondary chemical shift analysis on the GPB_**L**_/pAA coacervates at [NaCl] = 0.4 M (red) and 0.1 M (blue). **a** 1D ^13^C direct excitation (DE) experiment for both GPB samples. Regions with carbon beta (C*β*), carbon alpha (Cα), and carbonyl peaks (CO) are indicated; **b** Deconvolution of C*β* and Cα regions; **c** 1D ^13^C cross-polarization (CP; top) and insensitive nuclei enhanced by polarization transfer (INEPT; bottom) spectra, showing rigid and mobile residues, respectively. Peaks are labeled according to the secondary structure: α-helix (αh), *β*-sheet (*β*s), and random coil (rc); contaminant and solvent peaks are marked with a star; **d** Bar-graph representing the percentage of secondary structure. The secondary structures are colored green for *β*-sheet, light blue for α-helix, and orange for random coil, as indicated. Error bars represent the standard deviation obtained from 100 Monte Carlo iterations of peak-fitting deconvolution applied to a single dataset^82^.

Having established that oligoalanines can crosslink the coacervates, we set out to find out their secondary structure in these crosslinks. For this, we recorded MAS ssNMR experiments on the GPB_L_/pAA coacervate. Because of our specific interest in the oligoalanine structure, ^13^C- and ^15^N- labeled oligoalanines were employed for the grafted peptide chains (see Methods and Scheme S1 for synthesis). Two coacervates, with NaCl concentrations of 0.1 and 0.4 M, were prepared and analyzed with ssNMR in a hydrated state to better understand the effect of electrostatic interactions on the conformation and interactions of oligoalanines. First, 1D ^13^C direct excitation (DE) experiments were measured to detect the oligoalanine signals (Fig. 4a and Fig. S10a). The two samples with high and low ionic strengths gave similar spectra, suggesting a similar peptide conformation, even though they had different salt content. In contrast, the HP + NG-Ala/pAA spectrum revealed a distinct alanine conformation (Fig. S10a). In both grafted spectra, only the (labeled) oligoalanine peaks are visible, while the signals from the polymer backbone are undetectable in the noise. Interestingly, in the grafted samples four different peaks arose from the C*β* methyl region (near 20 ppm), suggesting the presence of at least four different alanine conformations (Fig. 4a).

The alanine backbone (Cα and CO) peaks were detected near 50–60 ppm and close to 180 ppm, respectively (Fig. 4a). These signals also showed multiple peaks, confirming the conformational heterogeneity of peptides in the coacervate. All peptide peaks display a similar linewidth, in contrast to narrower peaks coming from the solvent (marked with * in Fig. 4). To dissect the alanine secondary structure, we performed peak deconvolution and secondary chemical shift analysis (Fig. 4b; Table S2)^60–62^. Analysis of the (more resolved) C*β* shift^63^ revealed four different species with *β*-sheet (green), α-helix (light-blue), and random coil structures (orange), where the *β*-sheet is the dominant species (Fig. 4b and Figure S10b). Both GPB_L_/pAA samples display *β*-sheets as dominant species, around 60%, and near 20% of the sample has α-helix and random coil conformation. Both the low and high salt concentration coacervates have a highly similar secondary structure composition indicating that the difference in electrostatic strength does not have a considerable effect on the alanine secondary structure composition (Fig. 4d).

To understand if alanines in these secondary structures are immobilized or flexible, cross-polarization (CP) and insensitive nuclei enhanced by polarization transfer (INEPT) MAS NMR experiments were performed (Fig. 4c; Fig. S10c and d). In the DE experiments discussed above, the peak intensity is (largely) independent of the sample mobility permitting a (semi) quantitative analysis. However, in CP and INEPT experiments one detects (enhanced) signals from only rigid or flexible molecules, respectively. This prevents rigorous quantitative analysis but does enable the characterization of molecular motion^64^. The CP spectrum in Fig. 4c (top) shows that the most immobilized secondary structures are the *β*-sheet and the α-helix conformations, while the random coil peak is not visible (and thus not rigid). The figure introduces secondary-structure-based identifiers (See also 2D ssNMR analysis below). To study the effect of grafting on the peptide conformation, a HP + NG-Ala_L_/pAA coacervate with 0.1 M NaCl was prepared and compared with the grafted sample. Based on the secondary chemical shift analysis, the non-grafted sample contains the highest amount of *β*-sheet conformation, around 80%, a negligible percentage of α-helix and a similar amount of random coil to the GPB_L_/pAA samples (Fig. 4d). Thus, interestingly, also the alanines in the HP + NG-Ala_L_/pAA sample are very rigid and *β*-sheet rich, with less *α*-helix secondary structure.

**Fig. 5:**
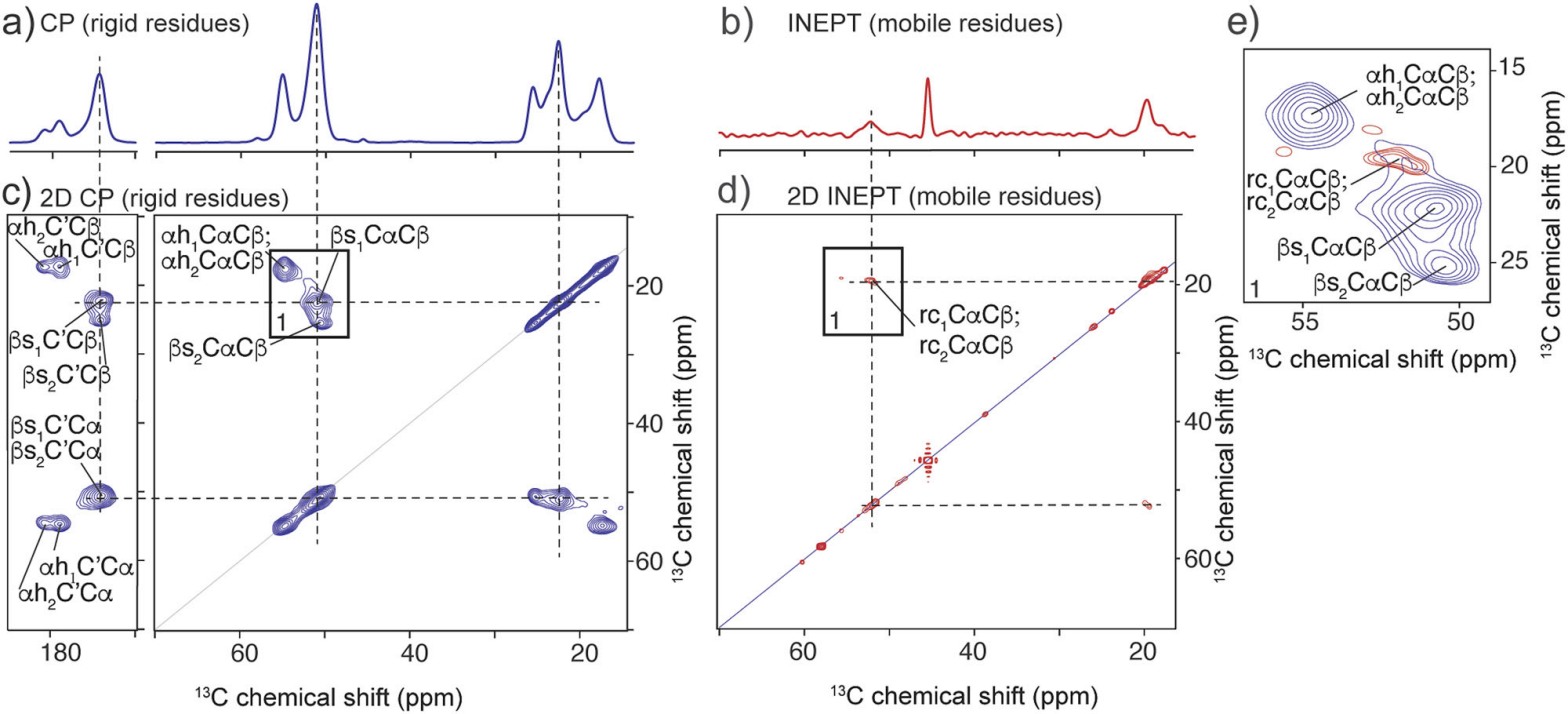
1D and 2D ^13^C MAS ssNMR experiments on the GPB_L_/pAA coacervate at 0.4 M NaCl. **a** 1D ^13^C ssNMR spectrum of the 2D CP-based experiment that shows rigid residues. **b** 1D ^13^C ssNMR spectrum of the 2D INEPT experiment, showing flexible residues. **c** 2D ^13^C-^13^C CP-based ssNMR experiment with 25 ms mixing time, showing rigid residues. Dotted lines show the intra-residue network of C*β*, Cα, and CO peaks of conformer *β*s_1_; **d** 2D ^13^C-^13^C INEPT-TOBSY ssNMR experiment showing mobile residues. Dotted lines show the intra-residue connection for rc_1_; **e** Spectral enlargement and overlay of the Cα region (black box in 2D spectra).

2D ^13^C-^13^C CP-based and 2D ^13^C-^13^C INEPT-based MAS ssNMR experiments were conducted to better understand the relationships between the observed signals, and to assign and identify the intra- and inter-residue connectivities (Fig. 5, S11, and S12). The 2D spectra resolve additional signals that overlap in the 1D spectra, revealing a further level of complexity: the presence of two types of *β*-sheet signals and two α-helix conformations (named *β*s_1_, *β*s_2_, αh_1_, and αh_2_). In addition, a CP-based 2D experiment showed an interaction between the two *β*-sheet signals (Fig. S12b and c), indicating that these two Ala conformations are in intimate contact. The observed signal intensity and polarization transfer time show that this contact has a maximum distance of 7 Å (Fig. S12b)^65,66^.

**Fig. 6:**
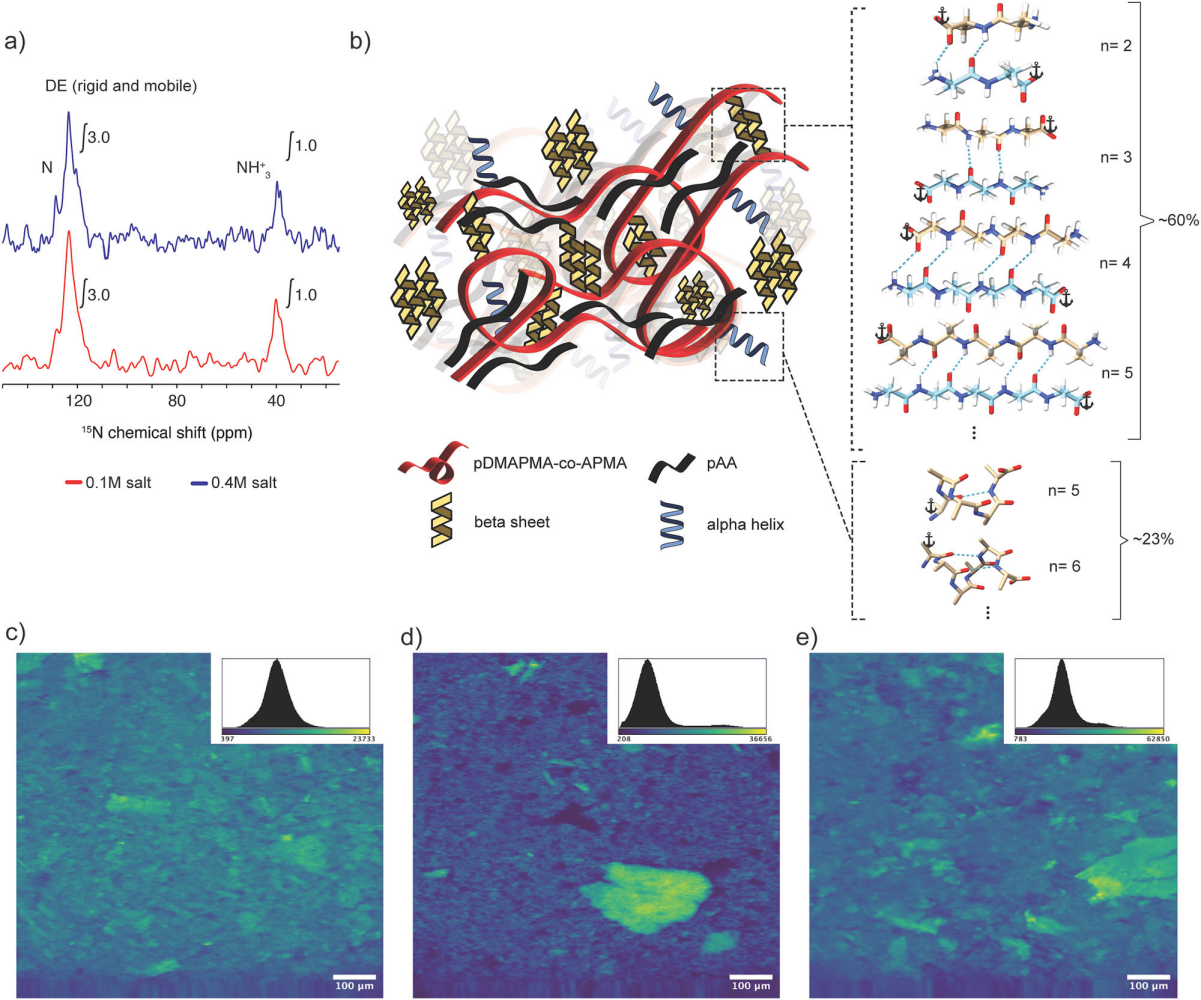
1D ^15^N ssNMR analysis, schematic model of alanines conformations, and localization of β-sheet aggregates within the coacervate matrix. **a** 1D ^15^N DE MAS ssNMR spectra on the sample with low (bottom; red) and high salt concentration (top; blue). In both spectra, the integrated peak areas suggest an average length of 4 alanines per peptide. **b** Schematic model of the GPB_L_/pAA coacervate (left) and alanines conformation models (right). According to the ssNMR, ~60% of oligoalanines form *β*-sheet structure, and 23% of oligoalanine form an *α*-helix structure. We propose that the secondary structure is partly determined by (variations) in peptide length (n), as indicated, with hydrogen bonding within an *α*-helix requiring more than five residues. The point where peptides are attached to the polymer chain is indicated by a black anchor symbol. ThT binding assay on **c** P-GPB/pAA, **d** HP* +* NG-Ala/pAA, and **e** GPB/pAA coacervates. All coacervates were prepared at 0.1 M NaCl and pH 7, in presence of 0.5 mM ThT. At least five micrographs were recorded for each sample. The images shown are representative of the complete set, and the insets are histograms showing the fluorescence intensity distribution of the pixels from all images. From the histograms, it can be observed that the coacervates of P-GPB/pAA show a unimodal distribution (corresponding to the relatively homogeneous background), while the coacervates of HP* +* NG-Ala/pAA and GPB/pAA show a second peak or shoulder at higher fluorescence intensity values. All scale bars are 100 µm.

1D ^15^N DE ssNMR experiments (Fig. 6a and S13a and b) showed two peaks, one representing N in a polypeptide backbone (around 120 ppm), and another from NH_3_^+^ (near 40 ppm), which must reflect the free peptide N-terminus. Peak integration was performed to estimate the relative amounts of the two signals as an indication of average peptide length (Fig. 6a and Fig. S13a). This analysis indicates an average of four alanines per peptide. In an attempt to connect these ^15^N signals to the distinct alanine conformers seen by ^13^C NMR, a 2D ^15^N- ^13^C CP-based experiment was done (Fig. S14). However, this spectrum suffered from a substantial ^15^N peak overlap of the ^15^N signals from the different conformers, limiting our ability to correlate variations in peptide length to different types of peptide conformations.

To summarize the ssNMR data, we proposed a schematic model for grafted alanines in the GPB_L_/pAA coacervate (Fig. 6b). The model shows that nearly 60% of the alanines are in a *β*-sheet conformation, while almost 23% have an α-helix conformation. As discussed in more detail below, we attribute at least part of the secondary structure variation to variations in peptide length. Likely, *β*-sheet formation is already effective for short peptides (≥2 alanine residues), while α-helix conformation is only possible for chains of at least five residues (the minimal length required to form an internal hydrogen bond)^67^.

We observed that the folded secondary structure is relatively rigid, even though the samples were measured in a hydrated and unfrozen state. The observed rigidity is most consistent with the presence of higher-order structures, mediated in part by hydrophobic interactions between helices and hydrogen-bonding between individual *β*-strands. These higher-order structures involving multiple peptides act as cross-links between polymer chains (to which individual peptides are attached) and thereby cause an improvement of the mechanical properties (Figs. 2e and 3). Interestingly, NMR shows that both α-helical bundles and *β*-sheet assemblies contribute to this peptide-based cross-linking mechanism. Based on ssNMR data, the oligoalanine peptides are not affected by salt concentration, neither in secondary structure nor in dynamics, so we hypothesize that the differences in mechanical properties between the coacervates at different salt concentrations are only caused by a change in the electrostatic interactions between the polymer backbone and pAA. Due to the time required for the preparation and analysis of ssNMR samples, we expect that the characterization described above corresponds to the equilibrated samples, in which aging has completely stopped. Interestingly, the fact that the peptide structure is virtually identical independent of the salt concentration confirms that salt concentration only affects the speed of the aging process, and not the equilibrium structure of the peptide-mediated crosslinks.

### Macroscopic distribution of oligoalanine aggregates

In addition to identifying the different secondary structures of the oligoalanine aggregates in our system, we used confocal microscopy to identify *where* those oligoalanines were located inside the polymer network. For this, we used Thioflavin T (ThT), a dye known to bind to peptide aggregates (particularly β-sheets), increasing its fluorescence when bound. We recorded confocal images of GPB/pAA, HP + NG-Ala/pAA, and P-GPB/pAA coacervates, observing an increase in fluorescence corresponding to *β*-sheet-rich aggregates in all samples (Fig. 6c–e). Between these samples, the P-GPB/pAA coacervate showed a more homogeneous distribution of *β*-sheet aggregates (Fig. 6c). On the other hand, the HP + NG-Ala/pAA showed fluorescent *β*-sheet aggregates on a relatively non-fluorescent coacervate background (Fig. 6d). Finally, the GPB/pAA coacervate showed a mixture of the two other structures: a homogeneous and highly fluorescent background, together with even more fluorescent *β*-sheet aggregates similar to the ones found in HP + NG-Ala/pAA (Fig. 6e). Confocal imaging of a GPB/pAA sample using pFTAA as a dye confirmed that the observed structures were β-sheet aggregates (Fig. S15), and not artifacts due to other type of inhomogeneities. Finally, we studied the morphology of the coacervates using SEM, detecting amorphous aggregates in both GPB/pAA and HP + NG-Ala/pAA samples (Fig. S16). Interestingly, fibrous aggregates could also be observed in the latter. We hypothesize that these aggregates cannot be formed in the GPB sample, as any structure that self-assembles in that case also needs to include the peptides that are grafted to the polymer backbone.

Combining the insights from ssNMR and confocal microscopy, we were able to propose structures for the different peptide-containing coacervates (Figs. 6b and S17). First, all of them contain oligoalanines of different lengths and with different secondary structures – mostly *β*-sheets, but with a considerable amount of random coils and *α*-helixes. Most peptide chains are aggregated, forming solid and rigid structures. However, these aggregates are distributed differently depending on whether they are grafted or not: grafted alanines form aggregates that are homogeneously distributed throughout the entire coacervate, while non-grafted ones form micrometer-sized aggregates, visibly separated from the coacervate matrix, and with a higher β-sheet content.

## Discussion

In this work, we have synthesized different spider silk–inspired peptide-polyelectrolyte conjugates, containing oligoalanine chains. In an analogous way to how spidroins undergo LLPS as part of their processing, we induced phase separation in our materials by addition of an oppositely-charged polyelectrolyte, forming viscoelastic coacervates. The grafted oligoalanine chains were able to crosslink the materials through peptide-peptide interactions, improving their mechanical properties at both low and high deformations. Specifically, even a small amount of grafted peptide chains (5% of monomers, in only one of the polyelectrolytes) increased their storage modulus by three orders of magnitude, and their work of adhesion in a tack test by a factor of five. This reinforcement was also sufficient to make the coacervates suitable for extrusion 3D printing, while in the absence of peptides the materials were too fluid and could not hold their shape.

Surprisingly, we found that oligoalanine chains had a strengthening effect even when they were not covalently linked to the polyelectrolytes. Through the formation of micron-sized β-sheet crystals that were physically embedded into the coacervate matrix, non-grafted oligoalanines improved the mechanical properties of coacervates to a similar magnitude to grafted ones. This effect, however, was restricted to low deformations, and outside of the linear viscoelasticity regime the material with grafted oligoalanines were much stronger than those with only physically embedded ones.

In addition to the increased mechanical properties, coacervates containing oligoalanine chains showed an unexpected behavior: salt-dependent aging. Aging is a common process in protein-based condensates, which frequently become more solid-like over time due to structural rearrangements of the protein structure. Unlike other metastable coacervates, aging in this case takes place due to the different kinetics of the two interactions involved in coacervate formation: when the two polyelectrolytes are mixed, the electrostatic interactions take place first, slowing down the formation of peptide-peptide crosslinks, which can take up to several hours to equilibrate. Increasing the salt concentration in this system (and thus weakening charge-charge interactions) resulted in coacervates that solidified faster, and not only in a loss of mechanical properties, as is typically the case in complex coacervates.

The material properties of the peptide-polyelectrolyte conjugates described in this work were not comparable to natural silks. This is not surprising, due to the differences in polymer length, composition, and the lack of processing of the synthetic materials, compared to the highly adapted spinning of silk. However, the results of these mechanical tests highlight how a few key elements, taken from the structure of spider silk (oligoalanine blocks acting as supramolecular crosslinks, possibility of LLPS), can be incorporated into a synthetic material, reinforcing its mechanical properties to a remarkable extent.

In addition to the mechanical study, we performed solid-state NMR analysis for a detailed characterization of the peptides in this material. The oligoalanines synthesized with this protocol are polydisperse, and their secondary structure depends on their length, and on whether they are grafted or not to the polymer chains. In particular, shorter chains and non-grafted chains almost exclusively form β-sheet-based assemblies, while longer chains and grafted chains also form α-helixes. In all cases, a small portion of the peptides is always present as random coils. CP and INEPT analyses showed that both β-sheets and α-helixes in our samples were in a rigid conformation, indicating their aggregation on a larger scale. This contrasts with the structure of spidroins, in which rigid aggregates incorporate almost exclusively peptides in a β-sheet conformation. Interestingly, there were practically no differences in secondary structure between coacervates at high and low salt concentrations: despite the differences in kinetics, salt did not affect the final structure of peptide crosslinks.

Combining these results with the rheological analysis, we conclude that oligoalanine grafting improves the mechanical properties of coacervates through the formation of aggregates, in which the oligoalanines adopt *β*-sheet and *α*-helical conformations. These aggregates serve as additional crosslinks between the polyelectrolyte chains, and are not affected by the salt concentration of the solution. Interestingly, crosslinking not only involves the peptides that are grafted to the backbone: free alanine chains can also participate in the formation of aggregates, and improve the mechanical properties of the material.

This is a proof-of-concept study, showing how peptide-containing coacervates can be used to understand different elements of spider silk. The synthetic route described here is easy to perform in only two steps from commercially available reagents, and yields large (gram-scale) quantities of polymer. Furthermore, it can easily be adapted to different polyelectrolyte chemistries, amino acid residues, as well as to different peptide lengths and grafting densities. This allows for a systematic parameter exploration in a way that is not practically feasible with natural silks: Using this protocol, multiple coacervates with different structures can be synthesized, studying how different structural parameters affect mechanical properties and peptide conformation. The results of such a study can be used as a base to understand similar structural variations between different silks.

Furthermore, the easy accessibility of these coacervates also allows for a thorough study of their processing. By using different extrusion techniques, as well as microfluidics, the coacervates described here can be spun into fibers in combination with different factors, such as salt gradients or shear forces. Spider silk has been described to form liquid-crystalline structures^37^, in a similar way to other systems that undergo LLPS^68^. In future research, we will investigate whether the coacervates described here show anisotropy, and if their processing can induce alignment. This can lead to insights on how natural silk spinning takes place, or to new principles for the processing of bioinspired materials.

## Methods

All materials and solvents were used as received from the manufacturer without further purification unless stated otherwise. N-[3-(Dimethylamino)propyl]methacrylamide (DMAPMAA), potassium persulfate, and sodium metabisulfite, labeled L-Alanine (99 atom % ^15^N, 99 atom % ^13^C), dioxane, sodium hydroxide (NaOH), and sodium bicarbonate (NaHCO_3_) were purchased from Sigma-Aldrich. N-(3-Aminopropyl)methacrylamide (APMAA) was purchased from TCI Europe (Zwijndrecht, Belgium). L-Alanine-NCA was purchased from Fluorochem (Hadfield, UK). Water was purified by reverse osmosis.

^1^H DOSY-NMR was performed on a 600 MHz spectrometer, using a temperature setpoint of 298 K, a z-gradient of 50 G/cm, and a stimulated spin echo (STE)-based pulse sequence. The gradient length (δ) was 1 ms, and its strength was increased linearly from 2 to 95% of the maximum gradient strength. The diffusion time (Δ) was set to 150 ms, sufficient to observe a signal attenuation of more than 90% for all small molecule signals (See Supplementary Note for more details). Other liquid-state ^1^H-NMR experiments were performed using a 400 MHz spectrometer. Chemical shifts are reported in parts per million (ppm), using tetramethylsilane (TMS; 0.00 ppm) as a reference.

### Size exclusion chromatography (SEC)

SEC was performed on a GPCMax system from Viscotek equipped with 302 TDA detectors array and two columns in series (PolarGel L and M, both 8 μm 30 cm) from Agilent Technologies. The columns and detectors were maintained at a temperature of 50 °C. DMF ( ≥ 99.9%, Sigma-Aldrich) containing 0.01 M LiBr was used as eluent at a flow rate of 1 mL min^-1^. Near monodisperse poly(methyl methacrylate) standards from Polymer Standard Services were used for the construction of a calibration curve. Samples were dissolved in the eluent at a concentration of ≈ 3 g L^-1^ and passed through a 0.45 μm PTFE filter prior to injection. Data acquisition and calculations were performed using Viscotek Omnisec software version 5.0.

### General synthesis

The following two reactions were performed to prepare ^15^N, ^13^C – labeled-L-Alanine-NCA, which was necessary to prepare the samples for ssNMR experiments (see below). A complete synthesis scheme with structures of all the synthesized compounds can be found in Scheme S1.

### Boc protection of ^15^N, ^13^C - labeled-L-Alanine

Boc-L-Alanine (^15^N, ^13^C), or Boc-L-Ala_L_, was synthesized following a previously reported method with some modifications^69^. Briefly, ^15^N, ^13^C - labeled-L-Alanine (1.00 g, 10.7 mmol) was dissolved in 11 mL of water, to which 22 mL of dioxane was added, resulting in a turbid suspension. Alanine was neutralized using 10 mL of NaOH 1 M, resulting in a clear solution again. After the alanine had dissolved completely, the reaction flask was cooled down with an ice bath and then NaHCO_3_ (0.94 g, 11.0 mmol) and di-tertbutyl dicarbonate (3.67 g, 16.8 mmol) were added to the solution as solids. The mixture was stirred overnight at room temperature, and concentrated in vacuo until approximately half of its original volume. Subsequently, 40 mL of ethyl acetate was added to the reaction crude and then the pH was decreased to 2.5–3 by slow addition of HCl 1 M under vigorous stirring. The product was extracted into 2 × 20 mL EtOAc. The combined organic phases were washed with 20 mL RO water, dried using anhydrous MgSO_4_, and concentrated in vacuo until a viscous oil was obtained. Pentane (20 mL) was added to the product, which was placed into a freezer (−20 °C) until crystallization. The desired product was isolated after decanting and drying in vacuo. (2.160 g, quant.) ^1^H-NMR (400 MHz, CDCl_3_): δ 4.97 (d, 1H, ^1^J_NH_ = 91.24 Hz), 4.32 (d, 1H, ^1^J_CH_ = 146.78 Hz), 1.44 (dm, 3H, ^1^J_CH_ = 129.94 Hz), 1.46 (s, 9H).

### Cyclization of ^15^N, ^13^C - labeled - alanine

^15^N, ^13^C-labeled N-carboxyanhydride Alanine (Ala-NCA_L_) was synthesized according to the procedure reported by Laconde et al., with some small modifications^70^. Typically, Boc-L-Ala_L_ (1.00 g, 5.18 mmol) was dissolved in 30 mL of ethyl acetate. T3P (3.78 mL, >50% weight, 6.42 mmol, 1.2 equiv.) was then added, followed by 0.420 mL of pyridine (5.20 mmol, 1 equiv.). The reaction was stirred for 2 hours at room temperature. Then, the crude was transferred into a separation funnel, and washed with 60 mL ice-cold water, and 2 × 10 mL ice-cold saturated NaCl solution. The organic phase was dried over anhydrous MgSO_4_, filtered, and concentrated in vacuo. The dried and still impure product was redissolved in a minimal amount of diethyl ether, and an equivalent volume of pentane was added. The solution was placed back into the rotavap, where the diethyl ether was carefully evaporated at 30°C until the first crystals of Ala-NCA_L_ were observed. The flask was then removed and placed in an undisturbed place until the crystallization was complete. Finally, the supernatant was decanted and crystals were dried in vacuo (0.097 g, 16%). ^1^H-NMR (400 MHz, CDCl_3_): δ 5.61 (dm, 1H, ^1^J_NH_ = 97.8 Hz), 4.41 (dm, 1H, ^1^J_CH_ = 146.6 Hz), 1.57 (dm, 3H, ^1^J_CH_ = 133.2 Hz).

### Polymer backbone synthesis

The polymer backbone (PB) (poly[(dimethylaminopropyl methacrylamide)-*co*-(aminopropyl methacrylamide)], or p(DMAPMAA-co-APMAA)) was synthesized through radical polymerization, following a protocol previously reported by us with some adjustments^52^. Here, 16.0 mL of DMAPMAA (15.0 g, 88.1 mmol) were dissolved in 170 mL of water under stirring, followed by the desired amount of APMAA-HCl (typically 0.790 g, 4.42 mmol, 0.05 equiv.). KPS (0.381 g, 1.41 mmol) and Na_2_S_2_O_5_ (0.083 g, 0.44 mmol) were added into two separate flasks and dissolved in 40 mL of water each. All three solutions were bubbled with nitrogen for 3 h to remove oxygen, and mixed under nitrogen flow. The resulting solution was stirred for three days, freeze-dried, and redissolved in around 40 mL of water. The polymer was then precipitated in around 500 mL of cold acetonitrile and dried in a vacuum oven at 50 °C overnight. To obtain a more easy-to-handle material, the resulting solid was redissolved in water, freeze-dried, and kept dry before further use. The resulting PB (2.46 g, 65%) was analyzed by ^1^H-NMR (Figs. S1 and S2) and SEC (Table S1).

A homopolymer containing only DMAPMAA (HP) was synthesized as a control following the same procedure, except for the addition of APMAA (Fig. S2).

### Grafting of oligoalanine chains through ring-opening polymerization

The final product was synthesized by grafting oligoalanine chains on PB, using NCA ring-opening polymerization. PB (4.00 g, 1.12 mmol of -NH_2_) was added to a flask along with the desired amount of Ala-NCA (typically 0.773 g, 6.72 mmol, 6 equiv.). The flask was sealed with a septum and dried through three cycles of N_2_ and vacuum. Subsequently, 60 mL dry DMF was injected into the reaction flask, and the mixture was stirred under a N_2_ atmosphere until the solids were completely dissolved. The reaction was then stirred vigorously at room temperature for three days, followed by precipitation in a mixture of hexane and diethyl ether (1:1 ratio, 1 L). The grafted polymer backbone (GPB) was isolated by filtration and dried in a vacuum oven at 50 °C overnight. GPB, as well as the controls (see below), were characterized using ^1^H-NMR and DOSY-NMR (Figures S3, S4, S5, and S6).

For the ssNMR samples, the same reaction was performed, but using the synthesized Ala-NCA_L_ (Ala labeled with ^13^C and ^15^N) instead of the commercial Ala-NCA.

The amine-initiated ring-opening polymerization of Ala-NCA can take place through two mechanisms^50^. Primarily, polymerization takes place through nucleophilic addition of the primary amine to the carbonyl group of the NCA-Ala monomers, resulting in the formation of chains that are grafted to the polymer backbone. However, in a competing mechanism, the primary amine acts as a base, deprotonating the monomer, which self-initiates the oligomerization. This second mechanism leads to the formation of oligomeric chains with a free C-terminus group, which are not attached to the polymer chain. To determine the effect of the grafted vs non-grafted chains on the mechanical properties of the final coacervates, we prepared two control samples: one with purified GPB (P-GPB), containing *only* grafted chains, and another one containing HP and *only* non-grafted oligoalanine chains (HP + NG-Ala) (See Scheme S1 for structures).

To prepare the P-GPB control, we dissolved GPB in a 1:1 mixture of water and DMSO and dialyzed it using a membrane with a cut-off molecular weight of 15 kDa (Repligen, USA). First, the polymer was dialyzed for 5 days against a 1:1 of H_2_O and DMSO, changing the water twice a day. This was followed by dialysis against pure water until no more DMSO was detectable in the external water by UV-Vis, and freeze-drying.

In order to prepare the HP + NG-Ala control, we repeated the grafting reaction with a mixture of Ala-NCA, HP and APMAA. Our aim was to maintain the conditions as similar as possible to the original reaction, but also ensure that the initiators for the ring-opening polymerization (primary amines) were not attached to the polymer chain. First, we transformed the commercial APMAA-HCl into its analogous amine, in order to ensure its solubility in DMF. To do this, we prepared a solution of APMAA-HCl in water and adjusted its pH to 13 using a 2 M solution of NaOH. The solution was extracted repeatedly with dichloromethane, and the organic phases were combined, dried with anhydrous MgSO_4_, and filtered. Dichloromethane was then evaporated by a gentle flow of N_2_, leaving APMAA as a colorless oil. Less than 24 h after preparation, APMAA was dissolved in dry DMF, mixed with DMAPMAA in a molar ratio of 0.05:1, and the rest of the reaction was performed following the same procedure as for the synthesis of GPB.

All controls were characterized using ^1^H-NMR (Fig. S5) and DOSY-NMR (Fig. S6).

### Formation of complex coacervates

All coacervates were prepared following the same protocol, independently of the polycation. First, we prepared 0.1 M stock solutions of GPB (or corresponding controls) and pAA, and adjusted their pH to 7 by using 1 M HCl. Then, we dissolved the required weight of NaCl into the pAA solution and added it to the solution of GPB to obtain a 1:1 charge ratio. Immediately after mixing the two solutions, the mixture was vigorously vortexed for 30 s to ensure homogeneous mixing, and then centrifuged at 4500 rpm for 15 min in order to separate the mixture into two phases: coacervate (bottom phase) and supernatant (top phase). The coacervate phase was kept in contact with its supernatant until use.

### Rheology

The mechanical properties of coacervates were measured by a rheometer (MCR302e, Anton Paar, USA). Frequency sweep measurements were conducted in the linear viscoelastic regime (LVR) from 100 to 0.1 rad/s at a constant strain of 1%. The time-dependent rheological properties of the samples were investigated by performing frequency sweep measurements every 30 minutes over 6 h for each sample. To avoid sample drying, the supernatant of each coacervate was poured around the measuring geometry and a homemade cap was placed on top to prevent water evaporation. All measurements were performed at a fixed temperature of 20 °C, using a 25 mm diameter cone-plate geometry with an angle of 1°.

### Probe tack test

The non-linear deformation of samples was measured by probe tack test, based on a protocol previously reported by our group^71^. The measurements were performed using the tack mode in a rheometer (MCR302e, Anton Paar, USA) equipped with a polished stainless steel bottom plate and a 10 mm diameter flat and sand-blasted stainless steel probe. Samples were placed in the center of the bottom plate and the probe was lowered to 250 µm. The probe was kept at 250 µm for 102 s before the start of measurement to ensure proper contact between the probe and the sample. After that, the probe was retrieved upward at a constant speed of 500 µm/s and the normal force and displacement were recorded. The stress and strain were calculated and stress-strain curves were plotted. The work of adhesion (W_adh_, the energy required to fully separate two surfaces far apart from one another) was calculated by measuring the area under the stress-strain curve. Finally, the maximum force to break and the work of adhesion of triplicate measurements were reported as average ± standard deviation.

### 3D printing

3D printing of inks was investigated using a 3D FELIX BIOprinter platform (IJsselstein, The Netherlands). First, complex coacervates were prepared from poly-acrylic acid and either PB or GPB, with a final concentration of 0.2 M (for each polymer, in charge units), and 0.1 M NaCl. Each coacervate was loaded by spatula into a syringe with a conical nozzle size of 0.41 mm, which was then closed by a plunger. It was not necessary to centrifuge the coacervates after loading them into the syringe to remove bubbles. Printing was performed inside a petri dish, fixed on the printing stage. The first layer was directly dispensed into the dish, and subsequent layers were deposited on top of the previous layer, alternating horizontal and vertical filaments. A total of five layers were deposited per sample. The following settings were used during printing: Extrusion multiplier = 2.5, Primary layer height = 0.2 mm, first layer speed = 100 mm/min. The geometry of all samples was a 1.5 × 1.5 cm square mesh, with a 2 mm inter-fiber distance.

### Solid-state NMR

The prepared material was packed into 3.2 mm zirconia thin-wall MAS rotors (Bruker Biospin, Billerica, MA) by pelleting the hydrated sample directly into the MAS NMR sample holder. The pelleting process was performed using a previously described sedimentation packing device under centrifugation at ~130,000 g in a Beckman Colter Optima LE-80K ultracentrifuge equipped with a SW-32 Ti rotor^72^. The excess water was removed, and an insert was introduced between the sample and the drive cap. The wet sample weight for each rotor was ~38 mg. Samples were studied by MAS ssNMR in a hydrated and unfrozen state. The spectra were acquired on an AVANCE NEO Bruker 600 MHz spectrometer equipped with a 3.2 mm HCN MAS probe with an Efree coil. 1D ^13^C INEPT^73^ and CP MAS ssNMR spectra were acquired under the following conditions: ^1^H 90° pulse was set to 2.5 μs corresponding to a rf power of ~100 kHz and ^13^C 90° pulse was set to 5 μs corresponding to a rf power of ~50 kHz; the CP step was performed with a contact time of 1 ms and using a 70 − 100% ramped-amplitude (RAMP) shape on the ^1^H channel and a 50 kHz square shape pulse on the ^13^C channel. During acquisition, the TPPM (two-pulse phase-modulated) decoupling scheme was employed using a pulse length for the basic decoupling unit of 5.8 μs at rf field strength of ~83 kHz^74^. The 1D ^15^N CP and DE experiments were recorded under the following conditions: ^1^H 90° pulse was set to 2.5 μs corresponding to a rf power of ~100 kHz, and ^15^N 90° pulse was set to 5 μs corresponding to a rf power of 50 kHz; the ^15^N CP step was performed using a 70 − 100% ramped-amplitude (RAMP) shape on the ^1^H channel and using a 50 kHz square shape pulse on the ^15^N channel. The 2D ^13^C-^13^C CP-based dipolar-assisted rotational resonance (DARR)^75^ ssNMR spectra were acquired using a 70–100% ramped ^1^H–^13^C CP step, and 3μs ^1^H and 5 μs ^13^C 90° pulses. Finally, ^1^H decoupling during acquisition time was ~83 kHz. The 2D ^13^C-^13^C total through bond correlation spectroscopy (TOBSY)^76^ experiments were recorded using ^1^H decoupling during acquisition time of ~71 kHz. These experiments combined refocused INEPT ^1^H − ^13^C transfers with 6 ms of P9^1^_3_ TOBSY (total through-bond correlation spectroscopy) ^13^C − ^13^C mixing^76,77^. The rotor-synchronized refocused INEPT technique requires slow T_2_ relaxation, enabling the identification of ^13^C − ^13^C correlations between highly mobile carbon atoms. The correlation between ^15^N and ^13^C was measured using the transferred-rotational echo double resonance (TEDOR) experiment^78^. 2D ^15^N-^13^C TEDOR spectra were recorded with a ^15^N 90° pulse of 5 μs. The TPPM ^1^H decoupling during TEDOR and acquisition was around 100 kHz. Additional experimental parameters for the ssNMR experiments are included in Tables S2 and S3 in the SI. The ^15^N and ^13^C ssNMR chemical shifts were indirectly referenced to liquid ammonia and aqueous DSS, respectively, by measuring adamantane ^13^C signals^79^. The ssNMR spectra were processed using NMRPipe^80^, and visualized using CcpNmr software version 2.5^81^. The 1D ^13^C DE experiments were deconvoluted in Dmfit using mixed Gaussian-Lorentzian functions to estimate the contribution of the different secondary structure elements^82^. The error analysis was also conducted in Dmfit using the Monte Carlo simulation approach^82^.

### Confocal imaging

The presence of *β*-sheet crystals in coacervates containing GPB and its corresponding controls was further investigated through adding ThT, a dye known to selectively label *β*-sheets, increasing its fluorescence in their presence^83^.

For this, a stock solution was prepared containing pAA (0.2 M), NaCl (0.2 M) and ThT (1 mM), and its pH was adjusted to 7 using a 1 M NaOH solution. Coacervates were prepared by mixing this solution with an equal volume of a stock solution containing GPB, P-GPB, or HP + NG-Ala (pH 7, 0.2 M in charge units). The coacervates were centrifuged, spread on a glass slide, and a few drops of supernatant were added on top of them to prevent drying. The prepared samples were measured immediately after preparation.

Confocal Laser Scanning Microscopy micrographs were obtained using a Zeiss LSM 780 confocal laser scanning microscope with an excitation beam of 440 nm, and a Zeiss 10X objective. The laser power, detector gain, dwell time, and pixel size were kept constant between experiments. Image analysis was performed with ImageJ, using the FIJI distribution^84^. At least five images were recorded for each sample.

As a confirmation, the same experiment was repeated for GPB/pAA coacervates, but using pFTAA as a β-sheet sensitive dye, in a final concentration of 3 µM. This experiment was performed using a Leica SP8 confocal microscope, with a 405 nm laser for excitation, and a 63x objective.

### SEM (scanning electron microscopy)

The surface morphology of the coacervate samples was observed using SEM (Nova NanoSEM650) at a working distance of 5 mm and an acceleration voltage of 5 kV. The sample preparation involved drop-casting 30 µL of liquid coacervate onto silicon wafers, followed by drying under ambient conditions. To avoid charging effects, the samples were coated with 10 nm gold prior to imaging.

### Statistical analysis

Statistical comparisons were carried out using an ordinary one-way ANOVA test with Tukey’s post-hoc test to assess differences between groups. P-values less than 0.05 were considered statistically significant (*p < 0.05, **p < 0.01, ***p < 0.001, ****p < 0.0001).

## Supplementary information

Supplementary Information

Description of Additional Supplementary Files

Supplementary Data 1

## Data Availability

The following files accompany the current study: - Supplementary Information (.pdf), containing the complete synthetic route and structures of all compounds, ^1^H-NMR and SEC characterization, DOSY ^1^H-NMR characterization, pictures of the prepared coacervates, additional rheology characterization, additional ssNMR data (^13^C and ^15^N chemical shift assignment and analysis; 1D ^13^C DE, CP, and INEPT MAS experiments; 2D ^13^C-^13^C CP-based DARR and INEPT-TOBSY spectra; ^13^C-^13^C CP-based DARR with different mixing times; ^15^N DE and CP MAS experiments; 2D ^15^N-^13^C TEDOR correlation spectra; detailed experimental conditions), confocal micrographs using pFTAA as a dye, SEM images, and a schematic representation of the structure of the coacervates prepared in this study. - Supplementary Data 1 (.pdf), containing ^1^H-NMR spectra of ^15^N, ^13^C-labeled tBoc-Ala and Ala-NCA. - Additional data supporting the findings of this study are available from the corresponding authors upon reasonable request.
