# Supplementary material for: Crosslinking, salt-induced aging, and secondary structure formation in Peptide-containing coacervates inspired by spider silk
Source: Commun Chem. 2025 Aug 28;8:264. doi: 10.1038/s42004-025-01634-8 (PMC12394569; doi:10.1038/s42004-025-01634-8)
Supplement: Supplementary file 1 — Supplementary Information [file 42004_2025_1634_MOESM1_ESM.pdf]

Supplementary Information

For

Crosslinking, salt-induced aging, and secondary structure formation in peptide-containing coacervates inspired by spider silk

Armin Amirsadeghi[1], Raffaella Parlato[2], Anna Kenbeek[1], Ana Rita Gaspar[1], Marta Oggioni[1], Alessia Lasorsa[2], Adrivit Mukherjee[1], Malak Jaber[3], Małgorzata K Włodarczyk-Biegun[1,4], Patrick C.A. van der Wel[2], Marleen Kamperman[1*], Guillermo Monreal Santiago[1,4*]

[1] Polymer Science, Zernike Institute for Advanced Materials, University of Groningen, Nijenborgh 3, 9747 AG, Groningen, The Netherlands

[2] Solid-state Nuclear Magnetic Resonance, Zernike Institute for Advanced Materials, University of Groningen, Nijenborgh 3, 9747 AG, Groningen, The Netherlands

[3] UMR7140 – Chimie de la Matière Complexe, CNRS, Université de Strasbourg, 4 Rue Blaise Pascal, 67081, Strasbourg, France

[4] Biotechnology Centre, The Silesian University of Technology, B. Krzywoustego 8, 44-100, Gliwice, Poland

* Corresponding authors

monrealsantiago@unistra.fr

marleen.kamperman@rug.nl

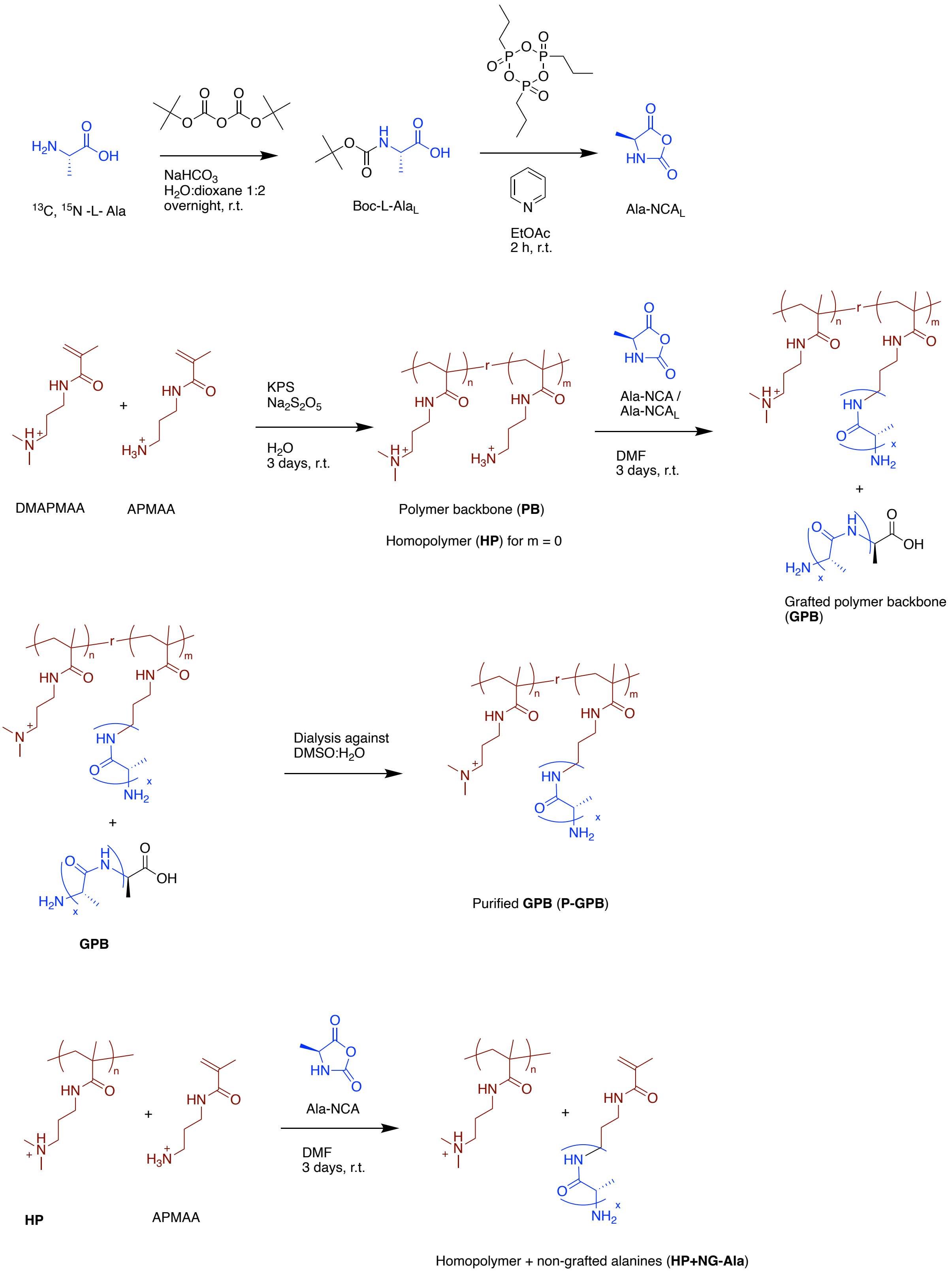

Scheme S1. Complete synthetic scheme, showing all reactions, products, and controls.

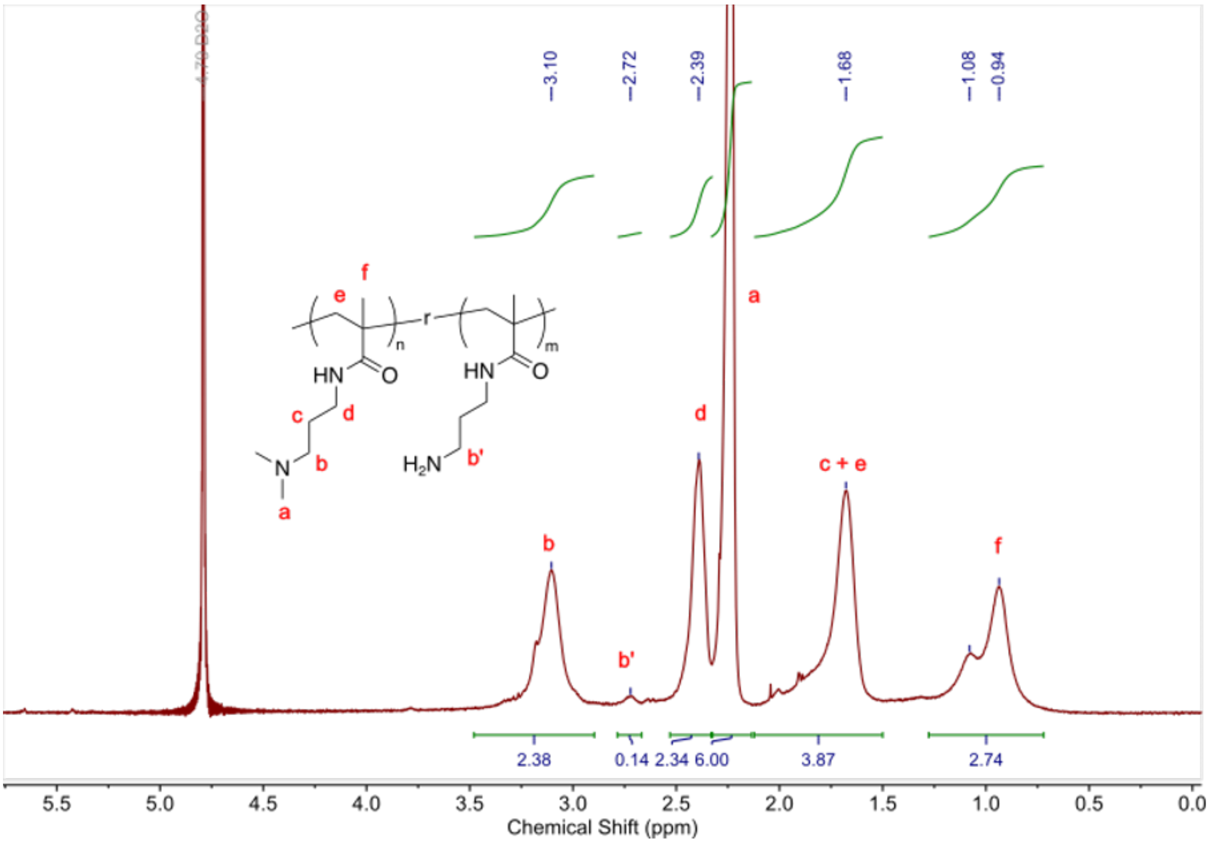

Figure S1. Structure and ^{1}H-NMR of the PB (D_2O, 600 MHz). In this case, the targeted m:n ratio was 5:100.

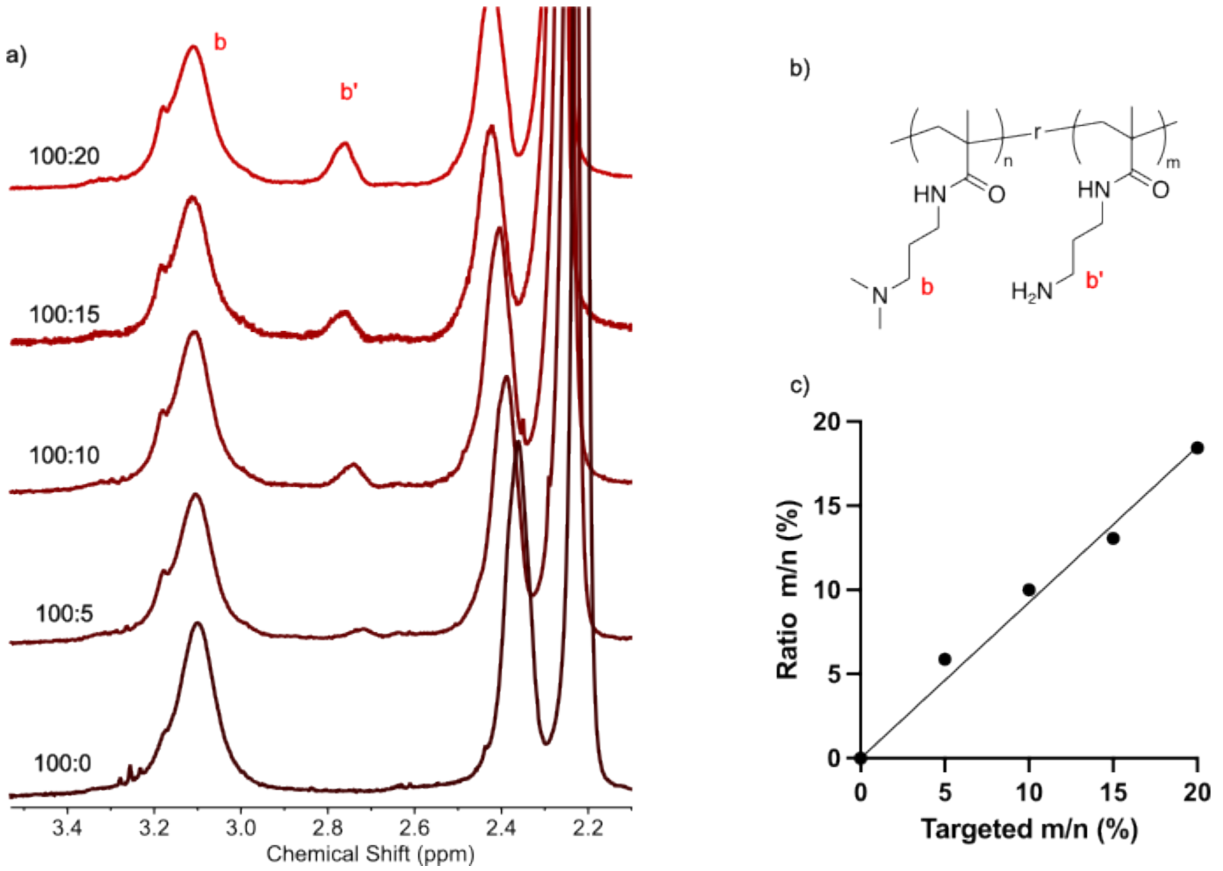

Figure S2. a) ^{1}H-NMR spectra of PB copolymers synthetized with different ratios between APMAA and DMAPMAA (m:n). The polymer that does not contain any APMAA (0:100) is referred to as HP (homopolymer) throughout the text. b) General structure of the copolymer, indicating the protons that are integrated to calculate the real ratio between the two monomers. c) Relationship between the real n:m ratio (calculated as the ratio between the two integrals) and the targeted one, showing that the amount of grafting points in the polymer can be reliably controlled during the synthesis.

Table S1: Molecular weights and polydispersity of different polymers, as calculated by size exclusion chromatography, showing that including APMAA in the polymerization does not significantly change the overall size of the backbone.

	M_n (* 10^3 g/mol)	M_w (* 10^3 g/mol)	PDI
**HP**	42	115	2.7
**PB (5 % grafting)**	40	134	3.3
**PB (10 % grafting)**	42	122	2.9

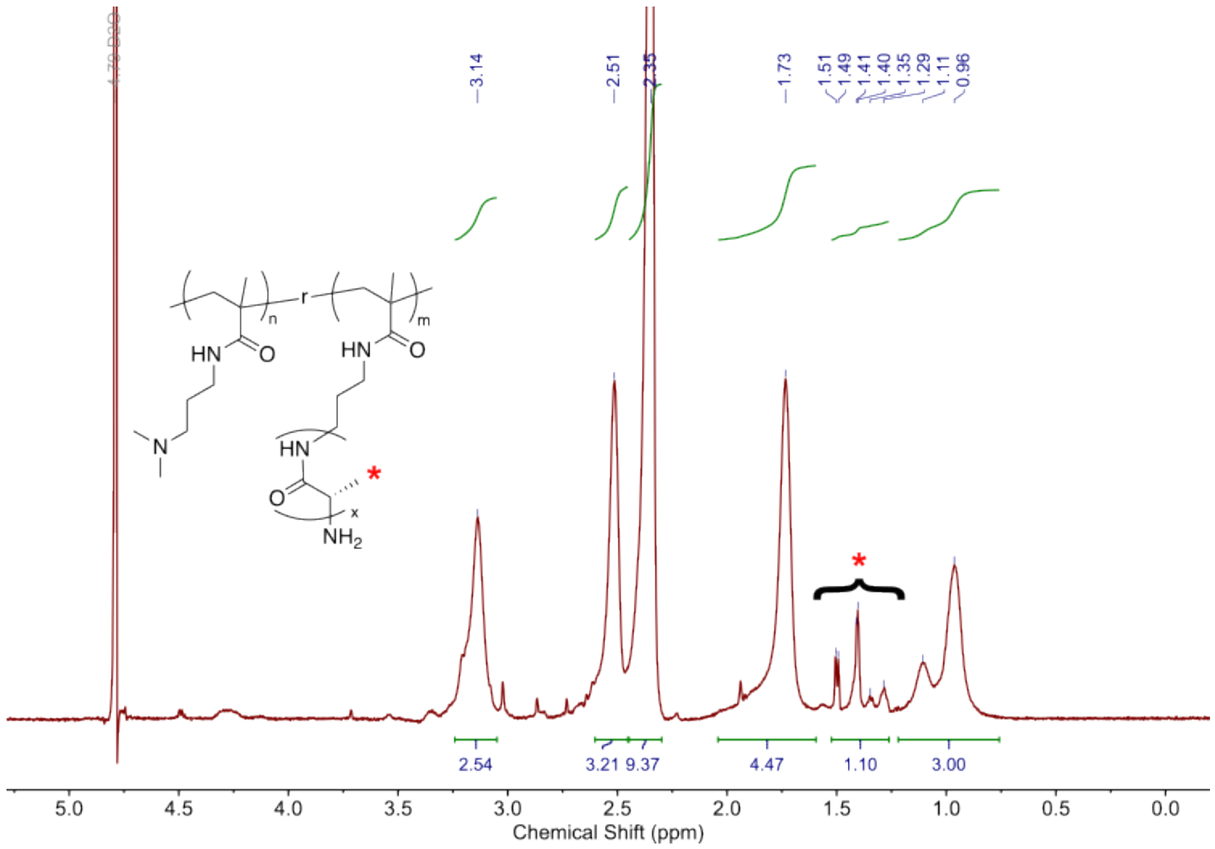

Figure S3. Structure and ^{1}H-NMR (D_2O, 600 MHz) of GPB, highlighting the signals that correspond to the side-chain CH_3 of alanine. These protons resonate as multiple signals due to the presence of grafted and non-grafted chains of different lengths (x) in the sample. The structure is drawn with only grafted oligoalanines for simplicity.

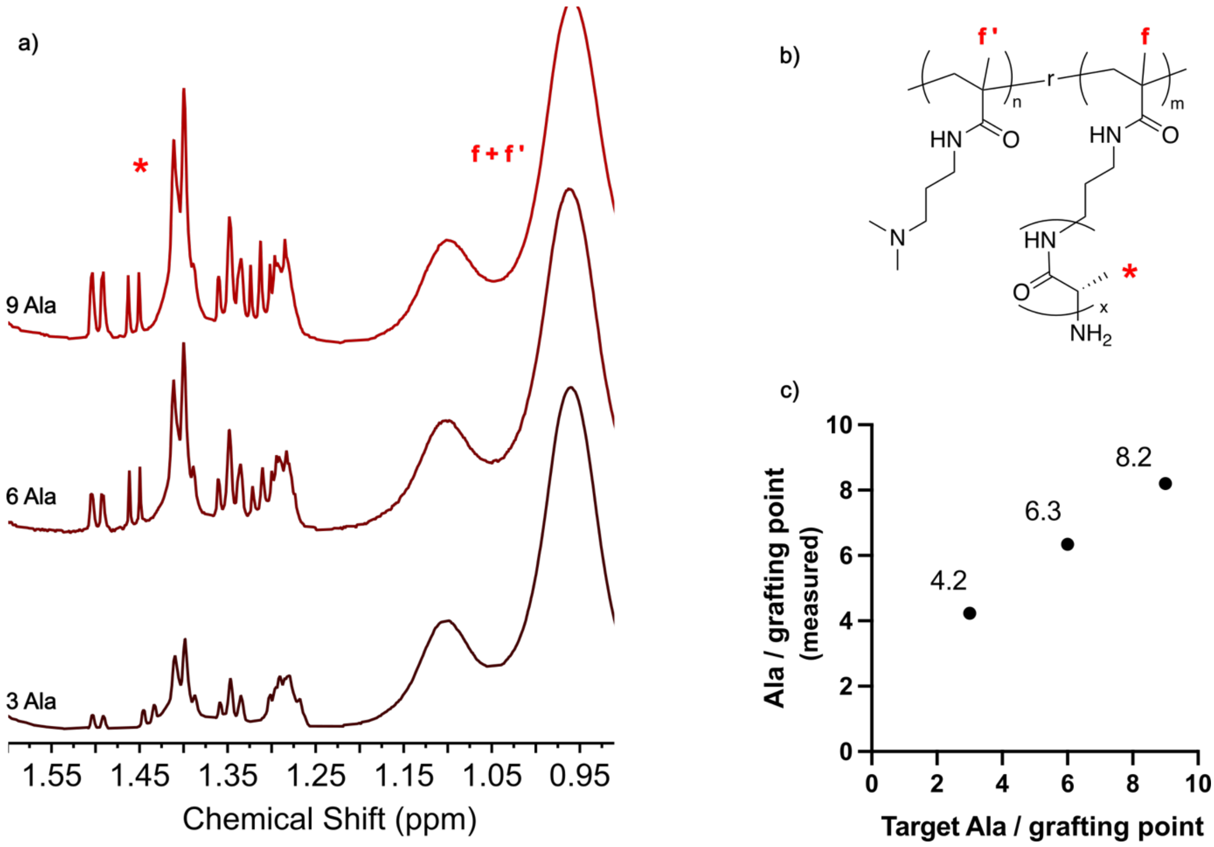

Figure S4. a) ^{1}H-NMR spectra (D_2O, 600 MHz) of GPB copolymers (m:n = 5:100) synthetized with different ratios between Ala-NCA and grafting points (m). b) General structure of the peptide-copolymer GPB (drawn with only grafted oligoalanines for simplicity). The protons that are integrated for the calculation of the number of alanines per grafting point are indicated. c) Relationship between the measured number of alanines per grafting point and the one targeted during synthesis. The number of alanines per grafting point was calculated from the NMR spectra, by comparing the integral of the alanine CH_3 protons (1.55-1.25 ppm, labelled as *) to the integral of the backbone CH_3 protons (1.2-0.7 ppm, f + f'), and dividing by the m/n ratio (see Figure S2). It should be noted that the signals between 1.55 and 1.25 ppm correspond to both grafted and non-grafted alanines, so the numbers in c) do not represent the average peptide length.

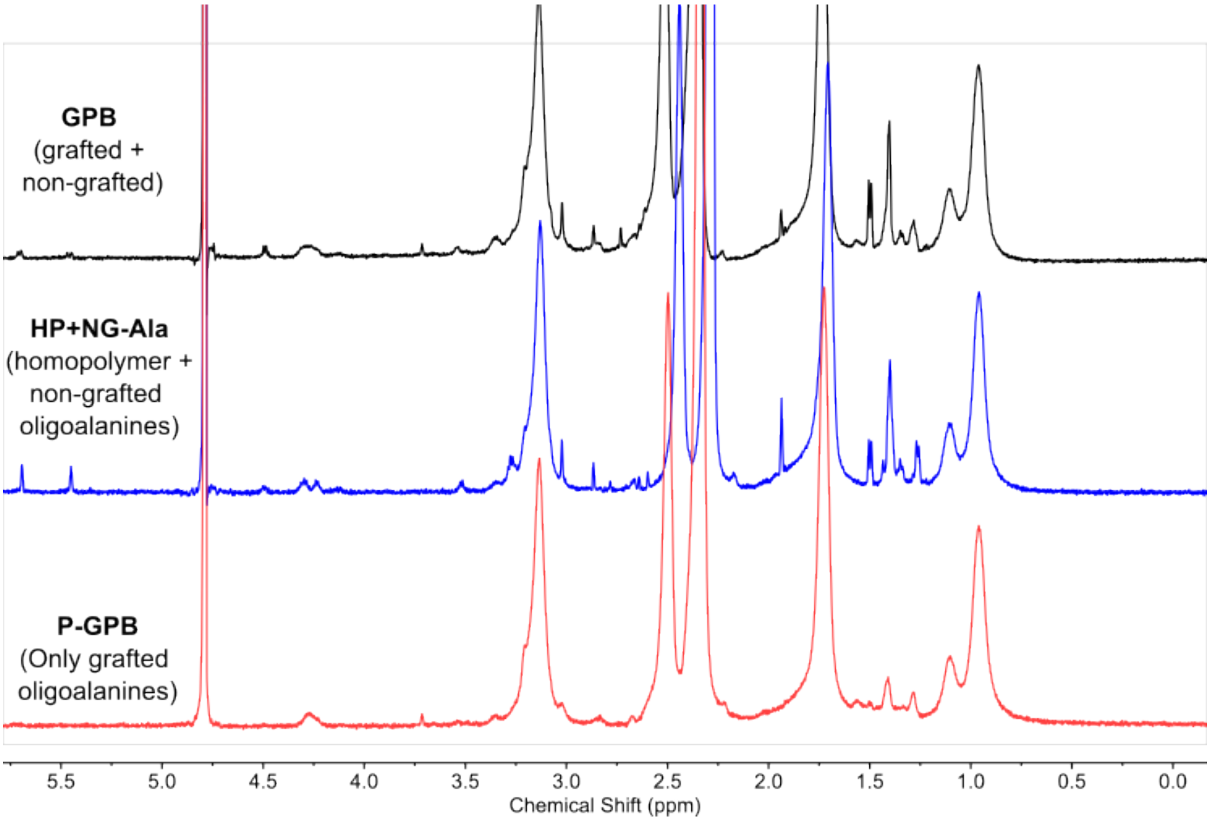

Figure S5. Comparison of the ^{1}H-NMRs of GPB, HP+NG-Ala and P-GPB. See Methods for the preparation of the three samples. An approximate ratio between grafted and non-grafted alanines of 3:1 was calculated by comparing the integral between 1.55 and 1.25 ppm for the GPB and P-GPB samples, using the CH_3 peak of the backbone (1.1-0.7 ppm) as a reference.

Supplementary Note: DOSY NMR

Here, we have used ^{1}H-DOSY-NMR to determine whether the oligoalanine chains synthesized in the ring-opening polymerization were grafted to the backbone of the polymer chain. DOSY-NMR is a technique that uses a characteristic pulse sequence, which includes a delay between excitation and refocusing. During this delay, molecules diffuse away from their original positions, leading to a decrease in the signal that depends on their diffusion coefficient: for small molecules, the signal decreases more, and for larger molecules, the decrease is smaller.[1] If two peaks have the same diffusion coefficient, it is assumed that their corresponding protons belong to the same molecule, or to molecules bound to one another.

The typical DOSY-NMR experiment involves three steps: 1) Recording a series of spectra using the DOSY pulse sequence and increasing gradient pulse strengths, 2) plotting the integral of each peak against the strength of the gradient, and 3) fitting the obtained data to calculate the diffusion coefficients, which are typically the only data reported. In our case, attempting to calculate diffusion coefficients from our data would not have been meaningful, since both the oligoalanine chains and the polymer backbone are polydisperse, and therefore all peaks represent an overlap of molecules of different lengths. For this reason, we have not performed this final calculation, and instead, we report here the "raw" integral data (Figure S6). These graphs already provide enough information to characterize the grafting of alanines in our samples:

For GPB (Figure S5c), the decrease of the alanine signals is clearly steeper than the decrease for the signal of the backbone – indicating that a number of oligoalanine chains are not attached to the polymer. However, at high gradient strengths, this decrease slows down, indicating the presence of a second, slow-diffusing population of oligoalanines – which are grafted into the backbone.

In the case of HP+NG-Ala (Figure S5d), the alanine peaks disappear much faster than the peaks of the backbone, indicating, as expected, that they are not covalently linked to each other. We also plot here the integral of the signals corresponding to the monomeric aminopropyl methacrylamide, which decrease at the same rate as the alanine signals. As expected, the oligoalanine chains are here grafted to this monomer.

Finally, for the P-GPB (Figure S5e), the decrease of the oligoalanine integrals overlaps with that of the backbone, indicating that they diffuse at the same speed, and therefore all alanines are grafted.

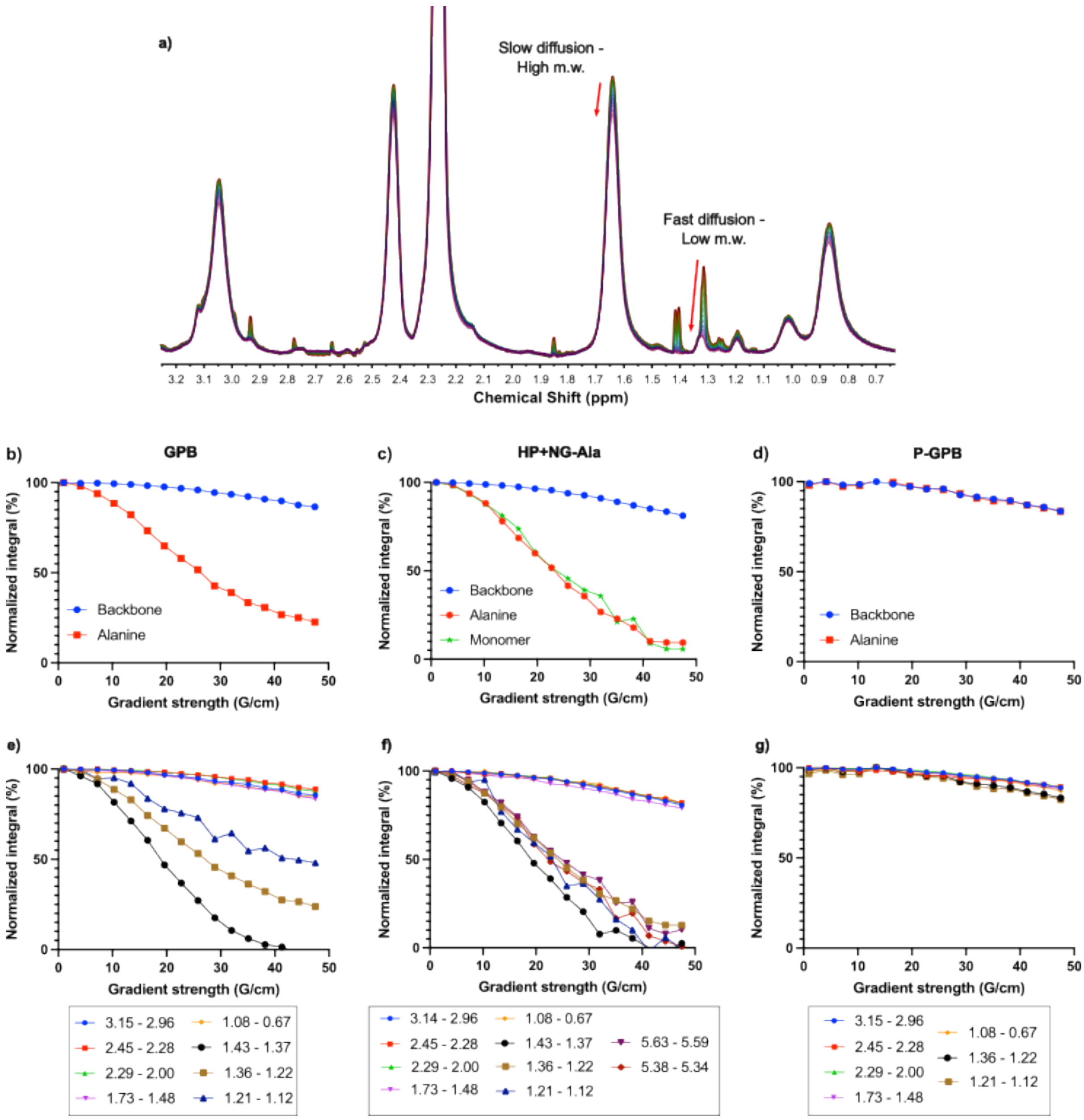

Figure S6. DOSY ^{1}H-NMR: a) Example of a series of spectra obtained in a DOSY ^{1}H-NMR experiment (in this case, corresponding to GPB). A slow-diffusing peak (1.7 ppm, corresponding to the backbone), and a fast-diffusing peak (1.3 ppm, corresponding to the side-chain CH_3 of alanine) are highlighted. b), c), and d) Evaluation of the integral of backbone and alanine peaks against the gradient strength for GPB, HP+NG-Ala, and P-GPB samples, respectively. See above for an assignment of backbone and alanine peaks. In c), the peaks corresponding to non-polymerized aminopropyl methacrylamide (5.6 and 5.4 ppm) are also shown, to highlight that in this case the oligoalanine chains are attached to this monomer. (e), f), and g) Integrals of each individual peak against gradient strength for these samples. The numbers in the legend represent the integration limits of each peak, in ppm.

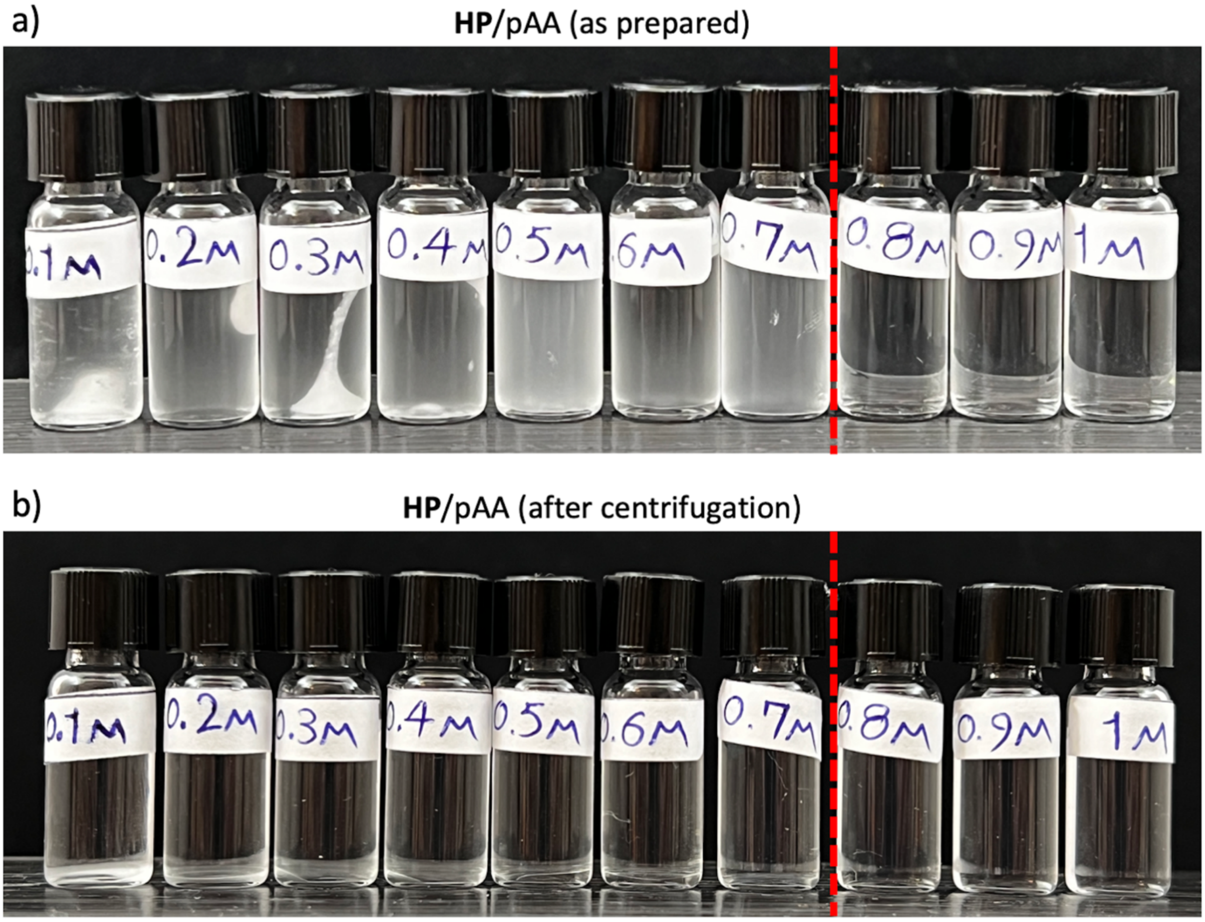

Figure S7. Appearance of mixtures of pAA with HP, at different [NaCl] concentrations (indicated in the labels on the vials), a) before and b) after centrifugation. Coacervates are typically micrometer-sized or larger, so the salt resistance (red line) was determined as the lowest concentration in which no scattering could be observed.

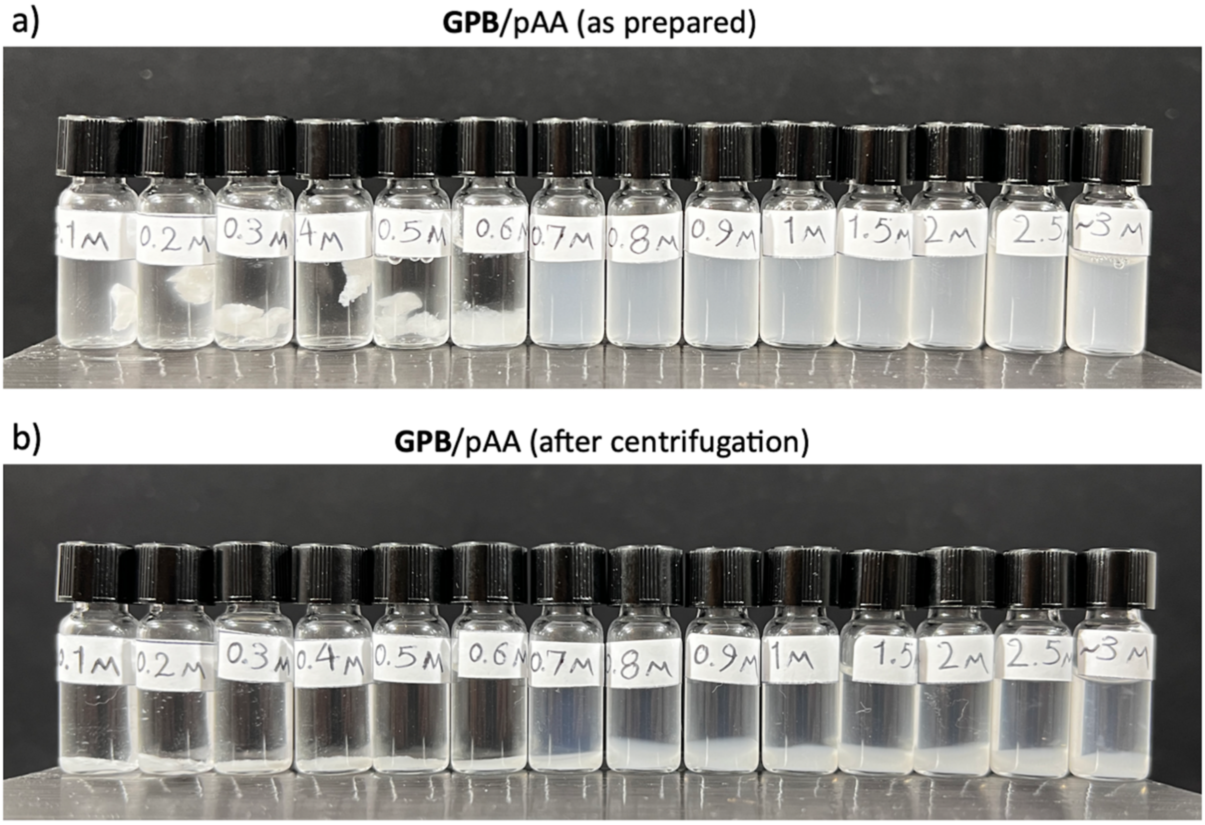

Figure S8. Appearance of mixtures of pAA with GPB, at different [NaCl] concentrations (indicated in the labels on the vials), a) before and b) after centrifugation. As it can be seen by the scattering of all samples, the system does not show a salt resistance and aggregation persists above 0.6 M. However, there is a clear morphological difference between samples under and over 0.6 M, which we ascribe to the disruption of electrostatic interactions through salt screening.

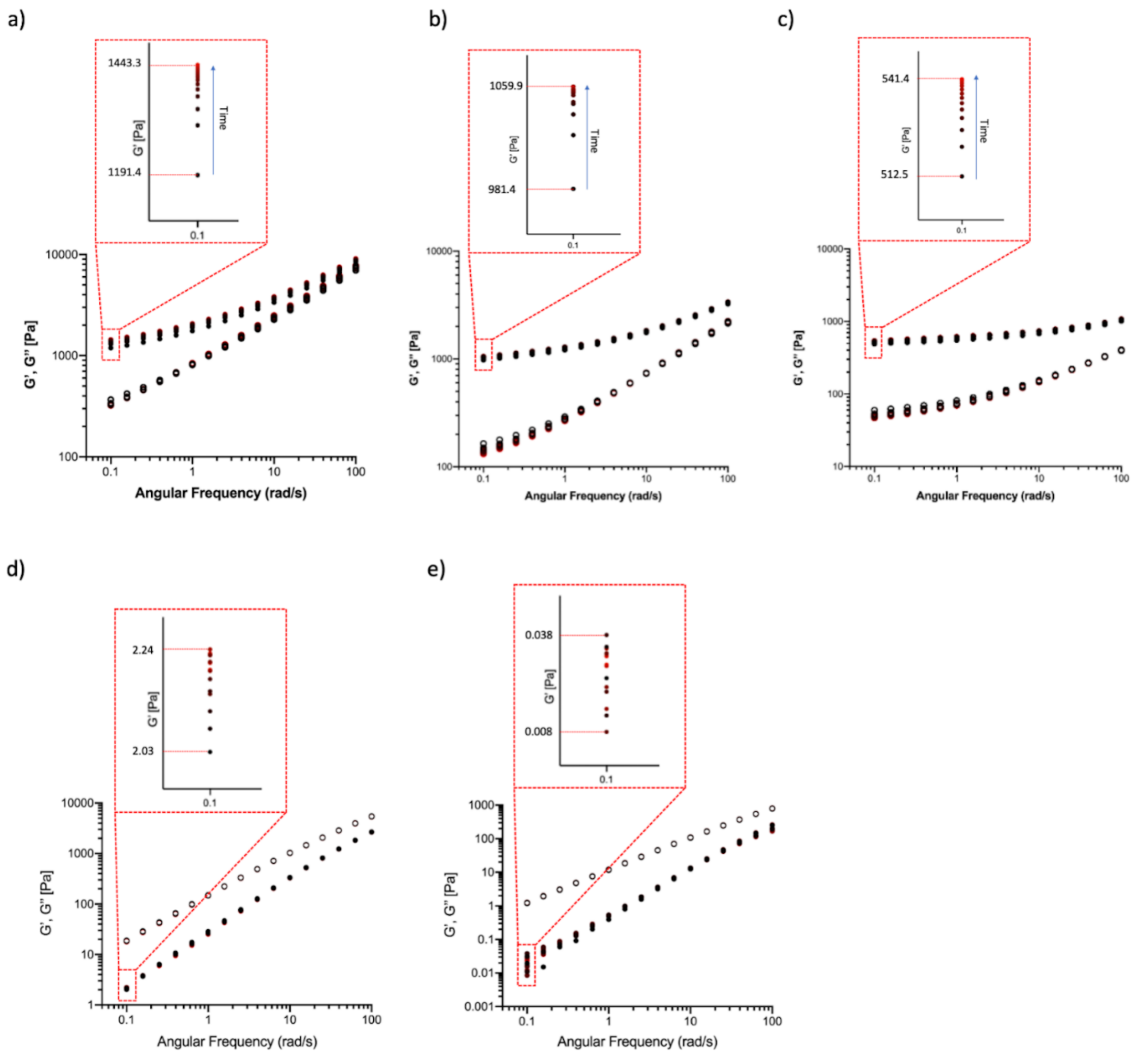

Figure S9. A sequence of 14 frequency sweep measurements performed every 15 minutes apart on GPB/pAA coacervates at a) 0.1, b) 0.4, and c) 0.6 M NaCl and HP/pAA coacervates at d) 0.1 and e) 0.4 M NaCl. pH was adjusted to 7 for all coacervates. The measurements were performed with a constant strain of 1% under linear viscoelastic regime.

Table S2. ^{13}C and ^{15}N chemical shifts (in PPM) of assigned residues in the coacervate samples and secondary chemical shift analysis for the secondary structure. The standard deviation of the ^{13}C of ^{15}N chemical shifts is ± 0.1-0.2 ppm based on the systematic comparison, during the peak assignment process, of peak positions between different spectra reported here (Table S4). The reported random coil values were subtracted from the measured chemical shift values[2] for the secondary chemical shift value (ΔCα-ΔCβ): strongly negative or positive values indicate β-sheet or α-helix structure, respectively. The positive sign in the last two columns indicates the presence of the residue in the indicated spectrum, noting an immobilized (CP) or flexible (INEPT) state, respectively. Overlapping peaks are marked with *.

	**Cα**	**Cβ**	**CO**	**N**	**ΔCα-ΔCβ**	**CP**	**INEPT**
**βs1**	50.9±0.2*	22.4±0.1	174.1±0.2*	122.8±0.1*	-5	+	
**βs2**	50.8±0.2*	25.3±0.2	174.1±0.2*	122.9±0.2*	-8	+	
**αh1**	54.9±0.1*	17.5±0.2*	178.9±0.2	125.4±0.2	3.9	+	
**αh2**	54.8±0.1*	17.5±0.2*	180.7±0.1	123.1±0.1	3.8	+	
**Rc1**	51.8±0.1	19.7±0.1*	174±0.1*	NA	-1.4		+
**Rc2**	52.7±0.1	19.5±0.2*	177.7±0.1	NA	-0.3		+

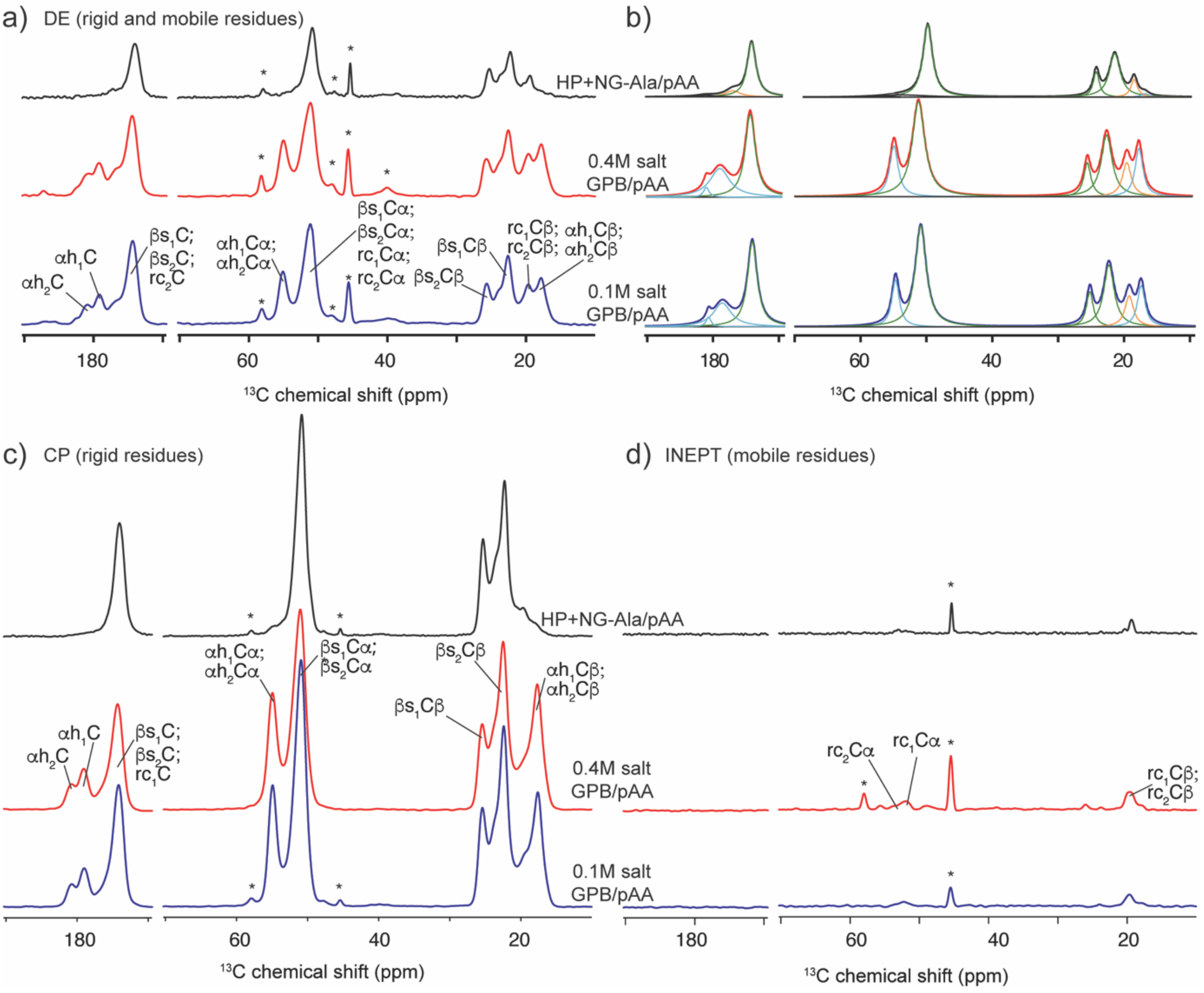

Figure S10. 1D ^{13}C DE, CP, and INEPT MAS ssNMR experiments recorded on coacervates with labeled oligoalanines. a) 1D ^{13}C Direct Excitation (DE) MAS ssNMR experiment on the HP+NG-Ala_L/pAA with 0.1 M NaCl; GPB_L/pAA with 0.4 M NaCl and GPB_L /pAA with 0.1 M NaCl, b) deconvolution of the ^{13}C 1D DE spectra. The fitted line for each peak is colored according to the secondary structure: β-sheet in green, α-helix in light blue, and random coil in orange, while the sum of the fitted peaks is shown in black for the non-grafted sample, in red for the 0.4 M NaCl, and in dark blue for the 0.1 M NaCl sample. c) 1D ^{13}C Cross Polarization (CP) MAS ssNMR experiment of the three samples, showing only the rigid or semi-rigid residues. The peaks are labeled according to their secondary structure α-helix (ah), β-sheet (bs), or random coil (rc); d) 1D ^{13}C Insensitive Nuclei Enhanced by Polarization Transfer (INEPT) MAS NMR experiment of the three samples, showing only signals from mobile residues. The HP+NG-Ala_L/pAA coacervate with 0.1 M NaCl is black, the GPB_L /pAA coacervate with 0.4 M NaCl is red, and the GPB_L /pAA coacervate with 0.1 M NaCl is dark blue. Contaminant peaks are marked with a star.

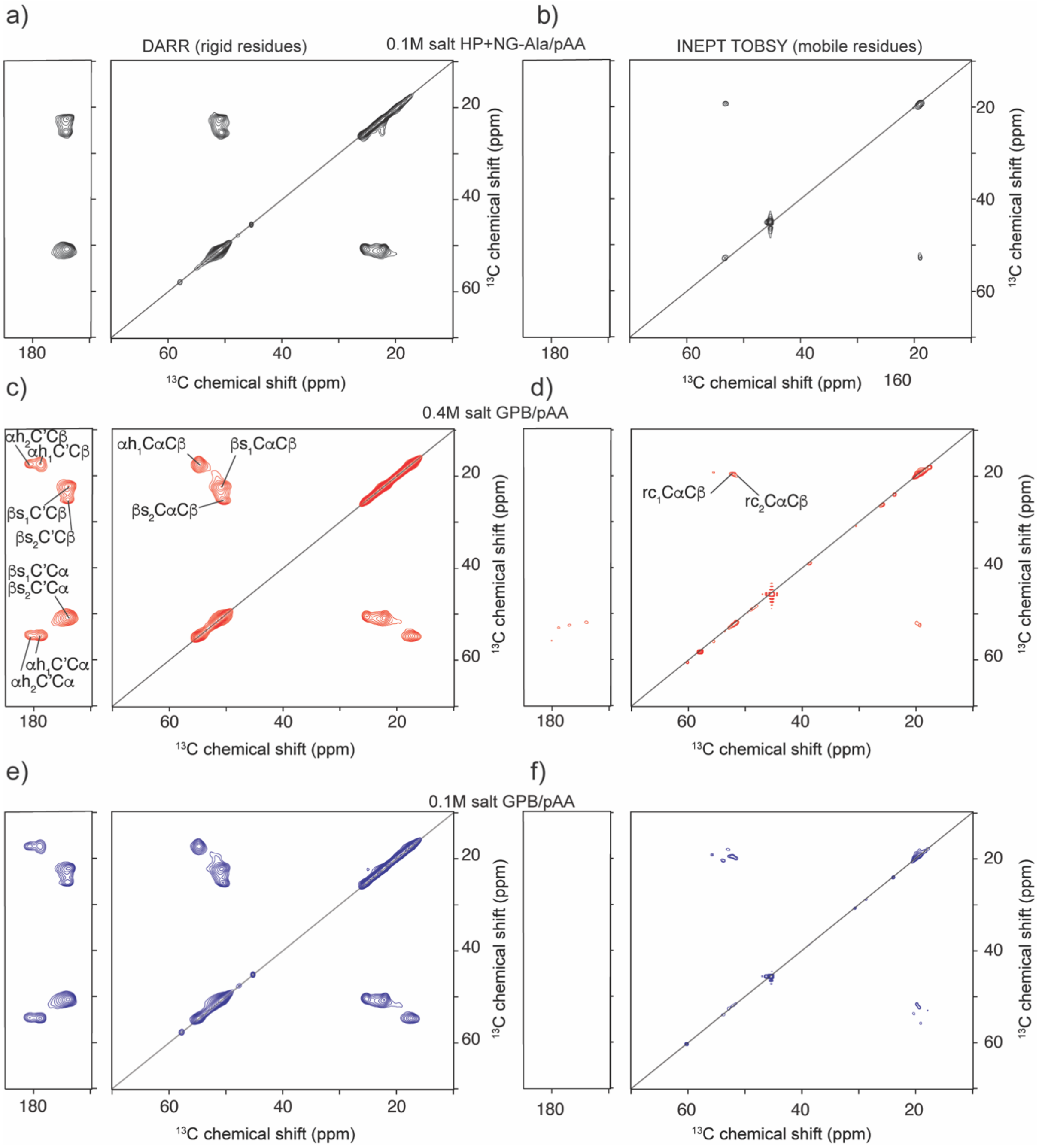

Figure S11. 2D ^{13}C-^{13}C CP-based DARR (left) and 2D ^{13}C -^{13}C INEPT-TOBSY (right) MAS ssNMR spectra for coacervates with labeled oligoalanine peptides. 2D ^{13}C -^{13}C DARR with 25 ms mixing time (left) and 2D ^{13}C -^{13}C INEPT-TOBSY (right) recorded on HP+NG-Ala$_L$/pAA coacervate with 0.1 M NaCl (a, b), GPB$_L$/pAA coacervate with 0.4 M NaCl (c,d) and GPB$_L$/pAA coacervate with 0.1 M NaCl (e,f). The CP-based DARR experiment shows only the signal from rigid residues, and with a mixing time of 25 ms, only intra-residue carbon connectivities are visible. On the other hand, the INEPT-TOBSY experiment shows the carbon connectivities from mobile residues.

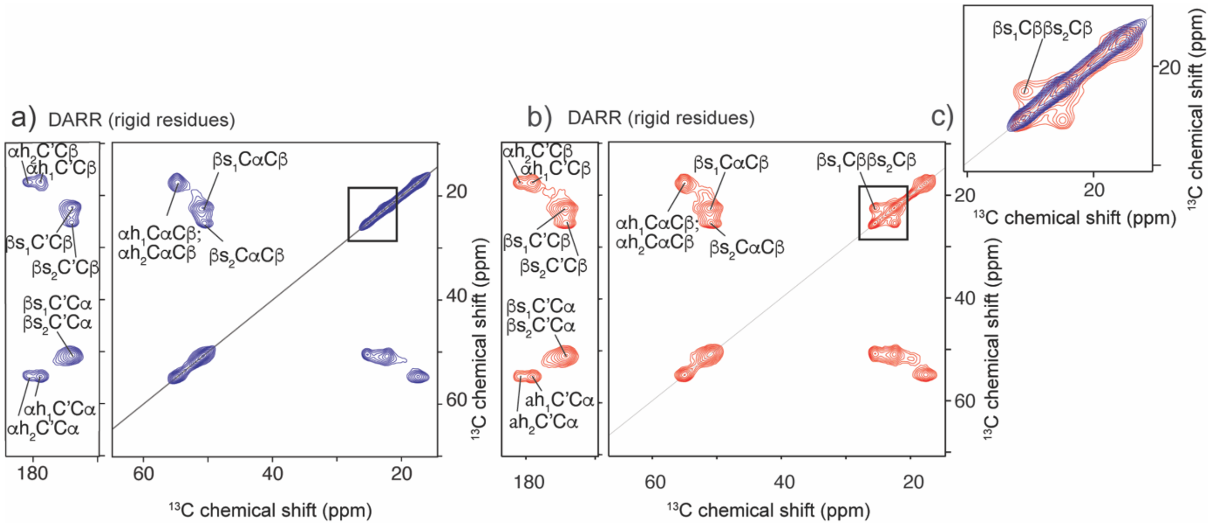

Figure S12. 2D ^{13}C-^{13}C CP-based DARR ssNMR spectra CP with 25 ms (left) and 200 ms (right) mixing time recorded on GPB$_L$ 0.4 M NaCl sample. a) 2D ^{13}C -^{13}C CP-DARR spectrum with 25 ms mixing time showing the intra-residue carbon network of rigid alanines (dark blue spectrum). The boxed area marks the spectral region for the Cβ of alanines featuring a β-sheet structure, which is enlarged in panel c. b) 2D ^{13}C -^{13}C CP-DARR with 200 ms mixing time showing both intra- and inter-residue carbon-carbon correlations (red spectrum). c) Enlargement of the Cβ region (~10-30 ppm) for both spectra overlayed and color-coded to highlight the observed interaction between the Cβ of two alanines, both featuring a β-sheet structure. This correlation shows that the two β-sheet structures are close in space. The mixing time is the time during which the magnetization is allowed to travel from a spin A to another spin.

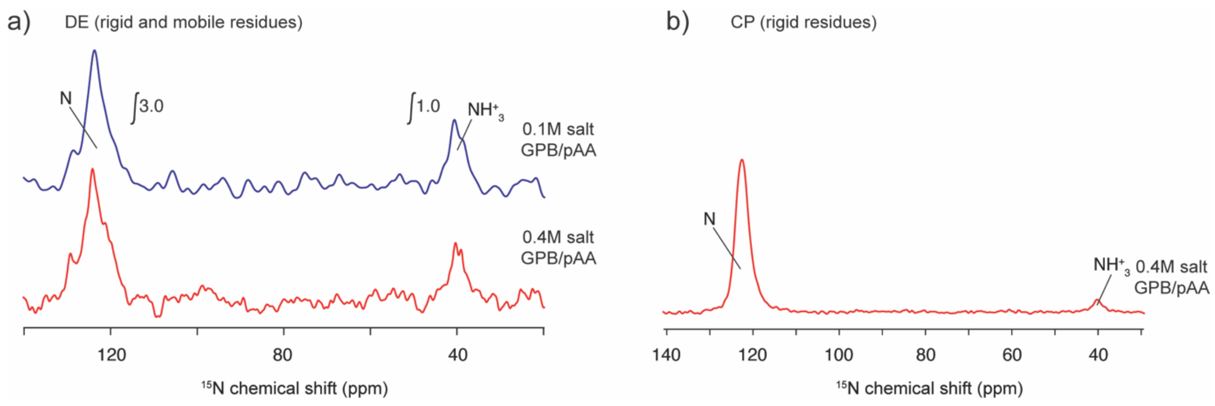

Figure S13. 1D ^{15}N DE and CP MAS ssNMR experiments on coacervates with labeled oligoalanines. a) 1D ^{15}N Direct Excitation (DE) MAS ssNMR experiment measured on the GPB_L/pAA coacervate with 0.4 M NaCl (red spectrum), and GPB_L/pAA coacervate with 0.1 M NaCl (blue spectrum). The integrated peak area is indicated in the spectrum for both N and NH_3^+ peaks. b) 1D ^{15}N CP MAS ssNMR experiments measured on HP+NG-Ala_L/pAA (black spectrum), GPB_L with 0.4 M NaCl (red spectrum) and GPB_L with 0.1 M NaCl (blue spectrum). The intensity ratio of the peak for the uncharged (peptide-bond) N, relative to the intensity of the charged NH_3^+ terminus, is higher than seen in the DE spectrum of panel a. This indicates a larger degree of dynamics of the latter, as well as a bias of longer peptides to form rigid supramolecular structures (compared to short peptides).

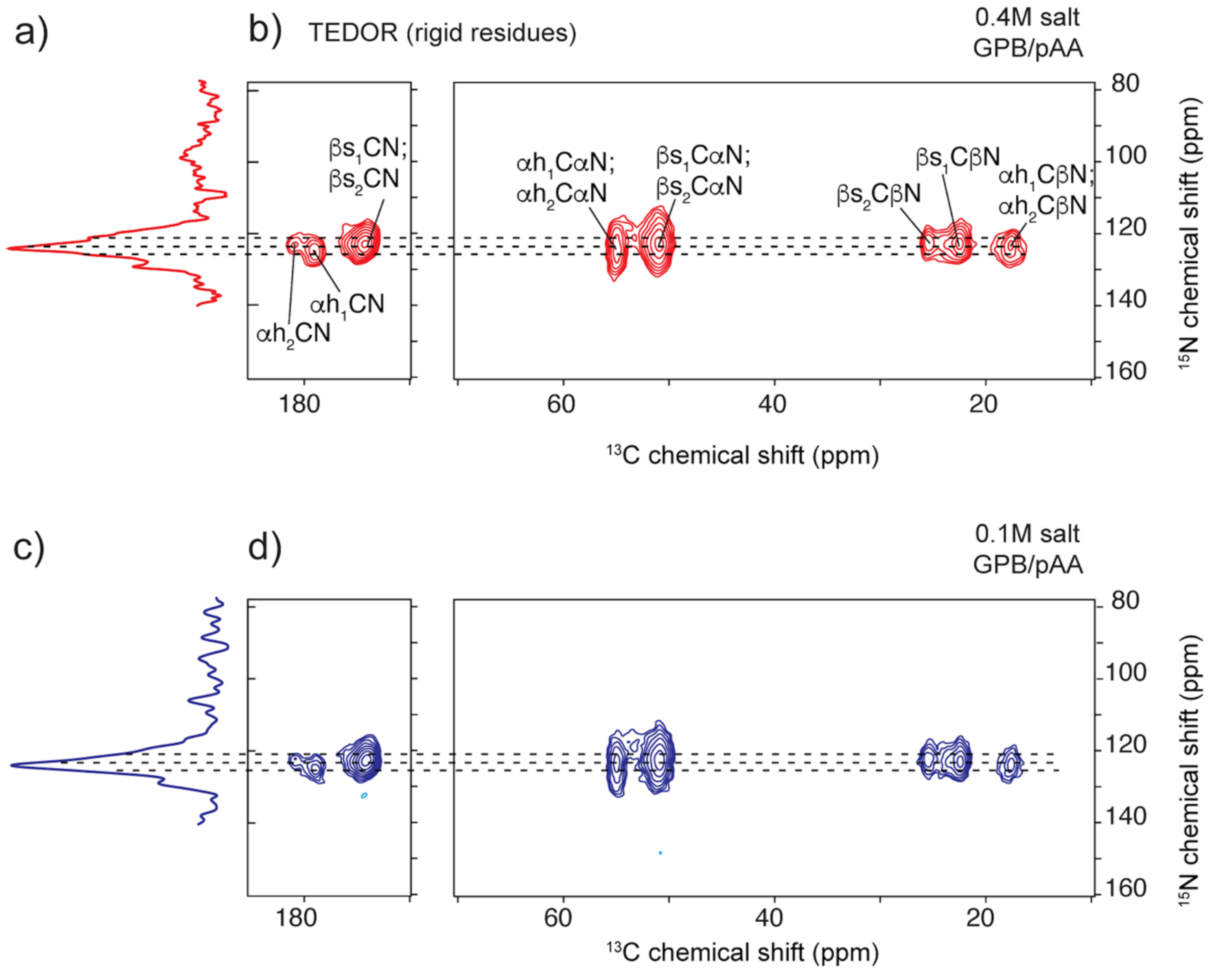

Figure S14. 2D ^{15}N -^{13}C Transferred-rotational Echo Double Resonance (TEDOR) correlation spectra of both labeled GPB_L samples. a) Enlarged spectral region of the 1D ^{15}N spectrum (80-140 ppm; see Figure S14), showing the signal from the nitrogen backbone of the grafted sample with 0.4 M NaCl; b) 2D TEDOR spectrum showing ^{13}C-^{15}N correlations in the same grafted sample; c) enlarged spectral region of the 1D ^{15}N (80-140 ppm), showing the signal from the nitrogen backbone of the grafted sample with 0.1 M NaCl; d) TEDOR spectrum showing the ^{13}C-^{15}N correlations in the same sample. Whilst the different conformers are distinguishable from their ^{13}C shifts, the ^{15}N signals overlap due to similar isotropic shift values and broadened linewidths.

Table S3. Detailed experimental conditions of 1D ssNMR experiments. Abbreviations: NS, number of scans per t_1 point; Temp., temperature; MAS, magic angle spinning rate; RD, recycle delay; TPPM, ^{1}H decoupling power during evolution and acquisition using the two-pulse phase modulation scheme; CT, contact time.

**Figure**	**Sample** [b)]	**Experiment**	**NS**	**Temp (K)**	**MAS (kHz)**	**RD (s)**	**TPPM (kHz)**	**CT (ms)**
**S11a**	HP+NG-Ala/pAA 0.1 M NaCl	^{13}C DE	256	278	13	3	83.3	NA
**4a; 5a S11a**	GPB/pAA 0.4 M NaCl	^{13}C DE	256	278	13	3	83.3	NA
**4a S11a**	GPB/pAA 0.1 M NaCl	^{13}C DE	256	278	13	3	83.3	NA
**S11c**	HP+NG-Ala/pAA 0.1 M NaCl	^{13}C CP	1024	278	13	3	83.3	1
**4c S11c**	GPB/pAA 0.4 M NaCl	^{13}C CP	1024	278	13	3	83.3	1
**4c S11c**	GPB/pAA 0.1 M NaCl	^{13}C CP	1024	278	13	3	83.3	1
**S11d**	HP+NG-Ala/pAA 0.1 M NaCl	^{13}C INEPT	256	278	13	3	83.3	NA
**4c, 5b, S11d**	GPB/pAA 0.4 M NaCl	^{13}C INEPT	256	278	13	3	83.3	NA
**4c S11d**	GPB/pAA 0.1 M NaCl	^{13}C INEPT	256	278	13	3	83.3	NA
**6, S14a, S15a**	GPB/pAA 0.4 M NaCl	^{15}N Hahn echo	16384	278	13	3	83.3	NA
**6 S14a,** S15c	GPB/pAA 0.1 M NaCl	^{15}N Hahn echo	16384	278	13	3	83.3	NA
**S14b**	GPB/pAA 0.4 M NaCl	^{15}N CP	2048	278	13	3	83.3	1

Table S4. Experimental conditions of 2D ssNMR experiments. Abbreviations: NS, number of scans per t_1 point; Temp., temperature; MAS, magic angle spinning rate; RD, recycle delay; TPPM, ^{1}H decoupling power during evolution and acquisition using the two-pulse phase modulation scheme; CT, contact time; Mixing, ^{13}C-^{13}C or ^{1}H-^{1}H mixing time (ms); t_1 evol., maximum t_1 evolution time expressed in number of t_1 points (real+imaginary) x t_1 increment time.

**Figure**	**Sample**	**Experiment**	**NS**	**Temp (K)**	**MAS (kHz)**	**RD (s)**	**TPPM (kHz)**	**CT (ms)**	**t_1 evol. (ms)**	**Mixing (ms)**
**S12a**	HP+NG-Ala/pAA	2D ^{13}C-^{13}C DARR	64	278	13	3	83.3	1	352x36	25
**5c, S12c, S13**	GPB/pAA 0.4 M NaCl	2D ^{13}C-^{13}C DARR	64	278	13	3	83.3	1	352x36	25;200
**S12e**	GPB/pAA 0.1 M NaCl	2D ^{13}C-^{13}C DARR	64	278	13	3	83.3	1	352x36	25
**S12b**	HP+NG-Ala/pAA	2D ^{13}C-^{13}C INEPT-TOBSY	48	278	10	2.5	71.4	2	488x24.5	NA
**5d S12d**	GPB/pAA 0.4 M NaCl	2D ^{13}C-^{13}C INEPT-TOBSY	48	278	10	2.5	71.4	2	488x24.5	NA
**S12e**	GPB/pAA 0.1 M NaCl	2D ^{13}C-^{13}C INEPT-TOBSY	48	278	10	2.5	71.4	2	488x24.5	NA
**S15b**	GPB/pAA 0.4 M NaCl	TEDOR	64	275	13	3	100	1.5	208x76.9	3
**S15d**	GPB/pAA 0.1 M NaCl	TEDOR	64	275	13	3	100	1.5	208x76.9	3

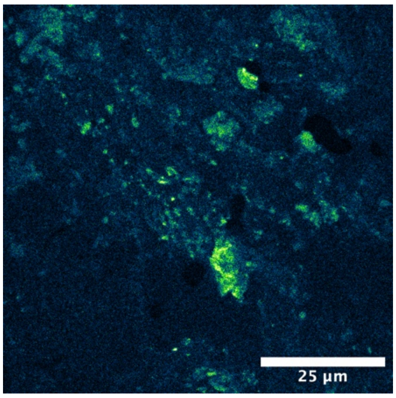

Figure S15. Confocal micrograph of a GPB/pAA coacervate, using pFTAA as a β-sheet sensitive dye. The coacervate was prepared with 0.1 M NaCl and pH = 7, and incubated overnight with 3 µM pFTAA.

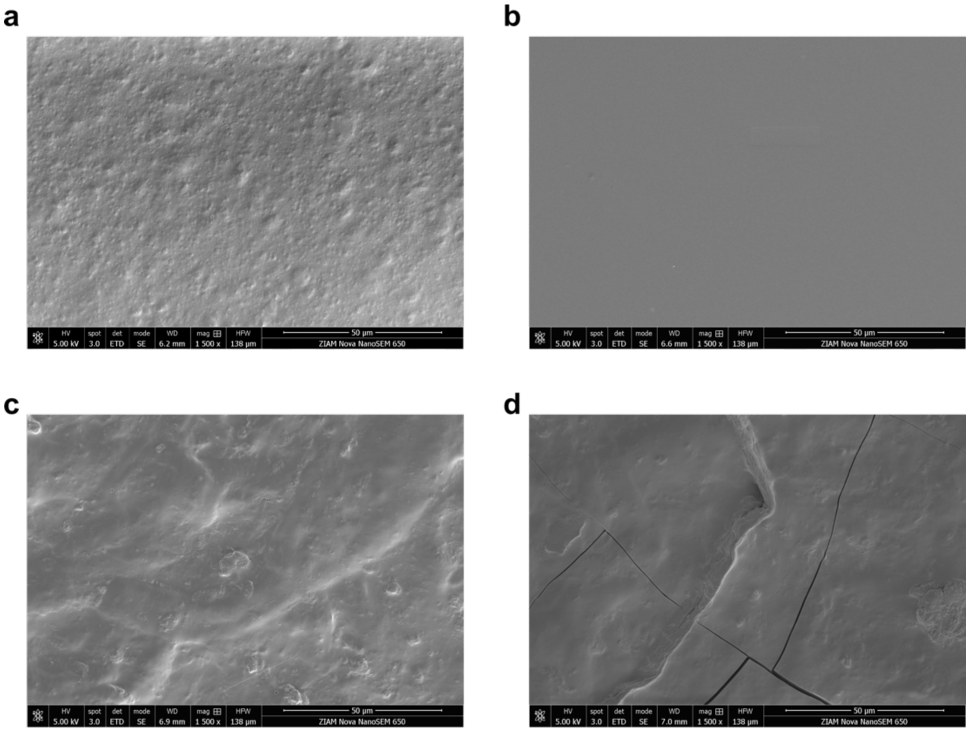

Figure S16. SEM images of different coacervate samples. a) GPB/pAA. b) HP/pAA c) and d): different regions of HP+NG-Ala/pAA. All coacervates were prepared with 0.1 M NaCl and pH = 7. Amorphous aggregates can be observed for the coacervates that contain oligoalanines, but not for the homopolymer. Furthermore, as observed in d), the HP+NG-Ala/pAA coacervates also contain fibrous aggregates.

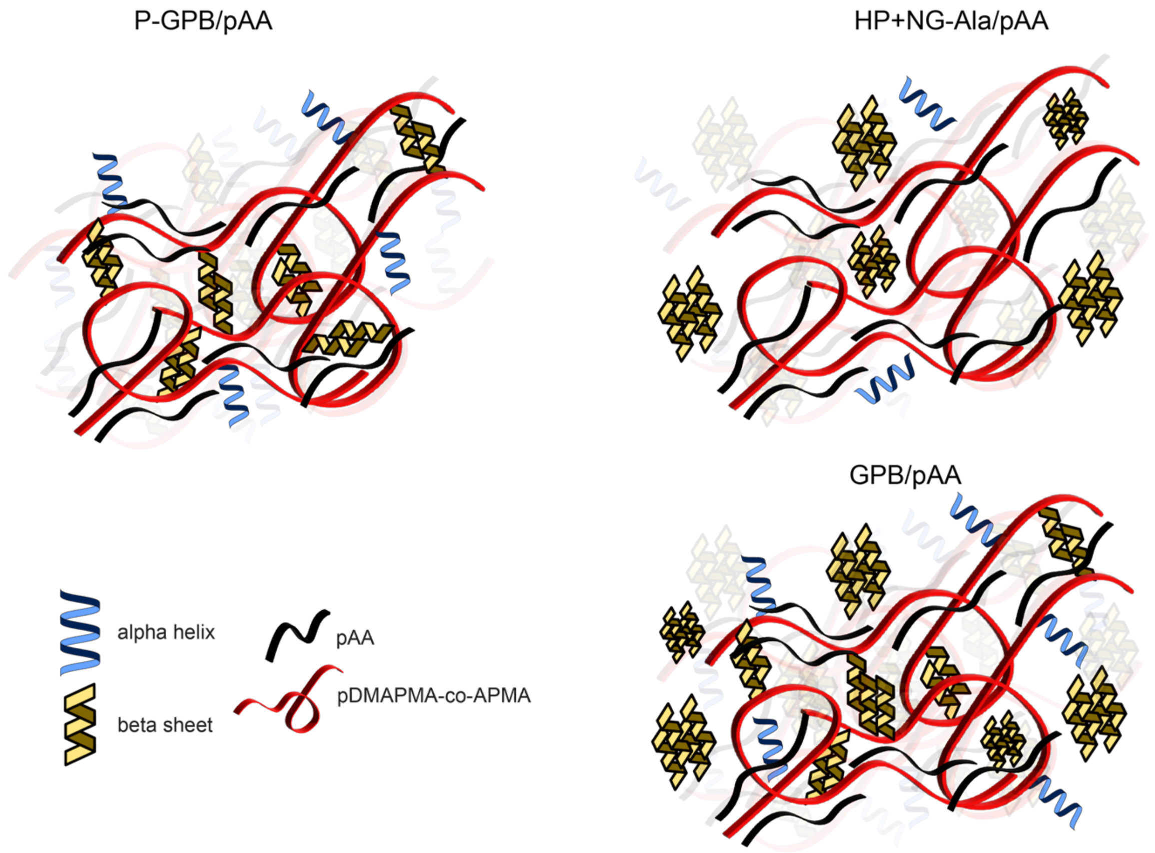

Figure S17. Schematic representation of the different coacervates prepared in this work, and the secondary structures observed in them. As deduced from ssNMR and confocal results, p-GPB/pAA coacervates contain grafted alanines, which form α-helixes and β-sheets. HP+NG-Ala/pAA coacervates contain mostly β-sheets, but those are separated from the rest of the polymeric structure, forming larger aggregates. Finally, GPB/pAA coacervates contain a mixture of all structures – α-helixes, β-sheets embedded in the coacervate matrix, and β-sheets separated from it.

Supplementary References

1. Johnson, C. S. Diffusion ordered nuclear magnetic resonance spectroscopy: principles and applications. *Prog. Nucl. Magn. Reson. Spectrosc.* **34**, 203–256 (1999).
2. Tamiola, K., Acar, B. & Mulder, F. A. A. Sequence-Specific Random Coil Chemical Shifts of Intrinsically Disordered Proteins. *J. Am. Chem. Soc.* **132**, 18000–18003 (2010).
