# Supplementary material for: Crosslinking, salt-induced aging, and secondary structure formation in Peptide-containing coacervates inspired by spider silk
Source: Commun Chem. 2025 Aug 28;8:264. doi: 10.1038/s42004-025-01634-8 (PMC12394569; doi:10.1038/s42004-025-01634-8)
Supplement: Supplementary file 2 — Description of Additional Supplementary Files [file 42004_2025_1634_MOESM2_ESM.pdf]

Description of Additional Supplementary Files

File name: Supplementary Data 1

Description: ^{1}H NMR spectra for ^{15}N, ^{13}C-labelled tBoc-Ala and Ala-NCA
