# Supplementary figures and images for: Crosslinking, salt-induced aging, and secondary structure formation in Peptide-containing coacervates inspired by spider silk
Source: Commun Chem. 2025 Aug 28;8:264. doi: 10.1038/s42004-025-01634-8 (PMC12394569; doi:10.1038/s42004-025-01634-8)

$^1\text{H}$  NMR ( $\text{CDCl}_3$ , 400 MHz)

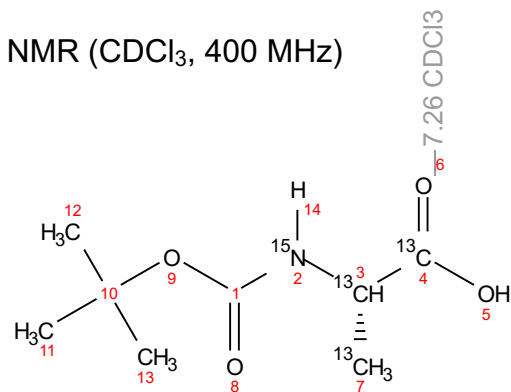

tBoc-Ala<sub>L</sub>

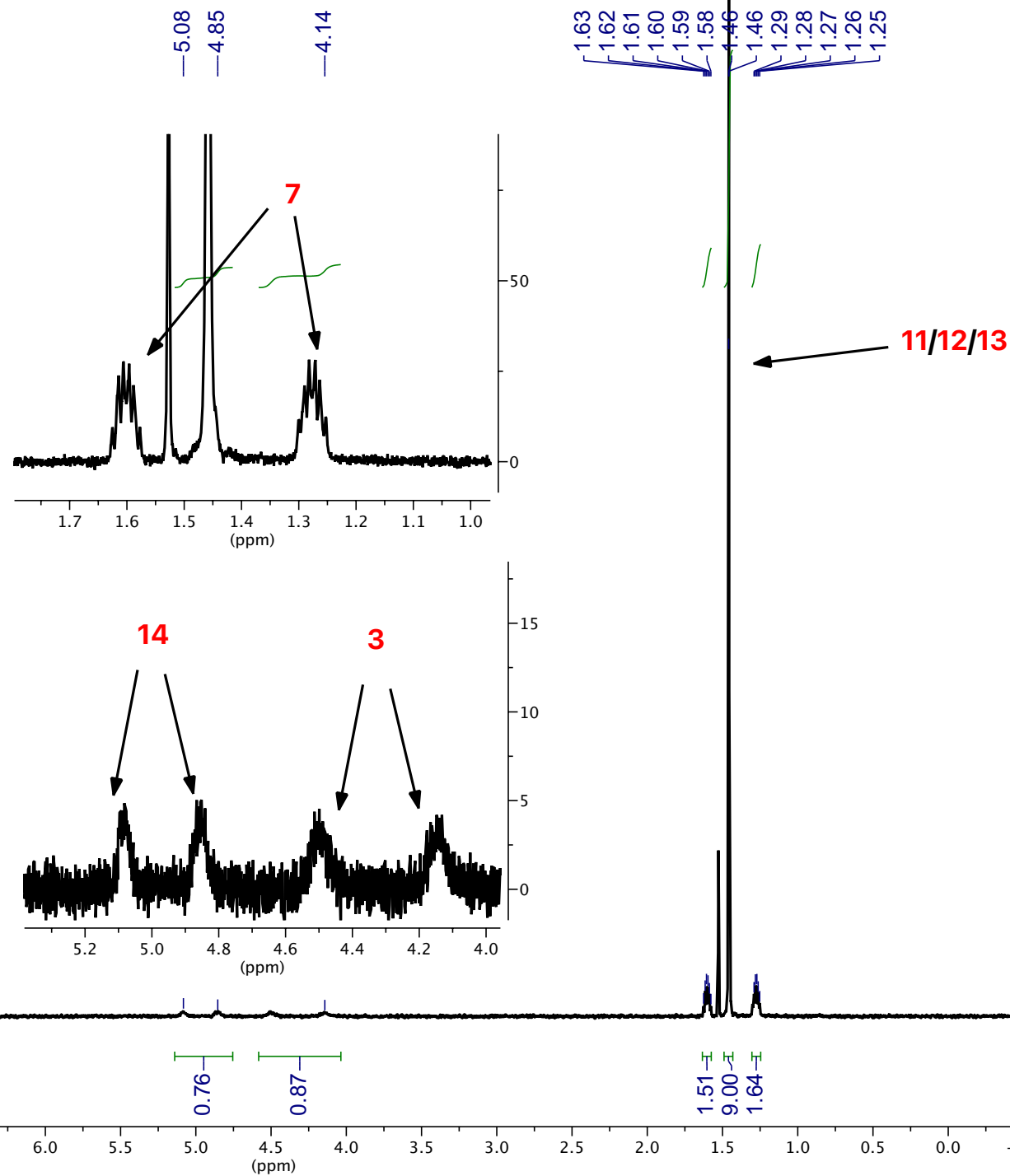

$^1\text{H}$  NMR ( $\text{CDCl}_3$ , 400 MHz)

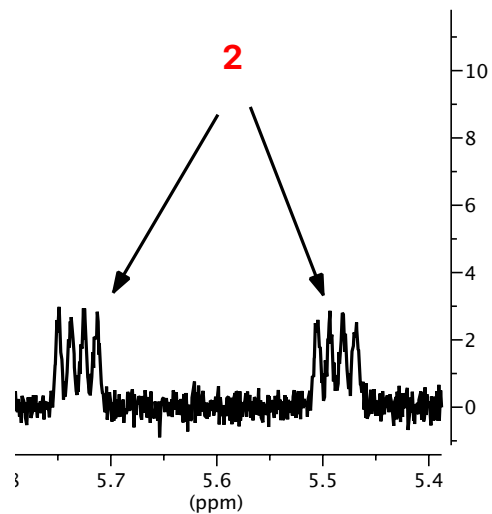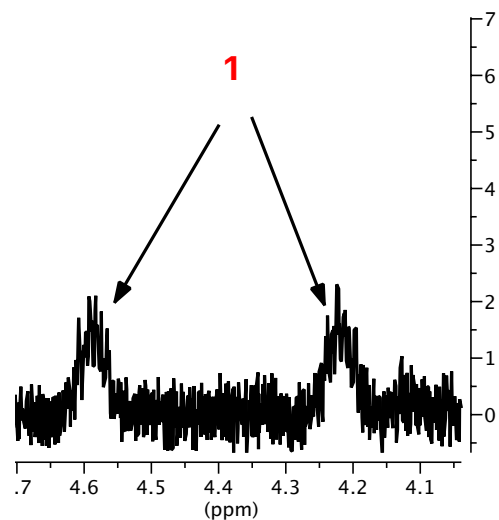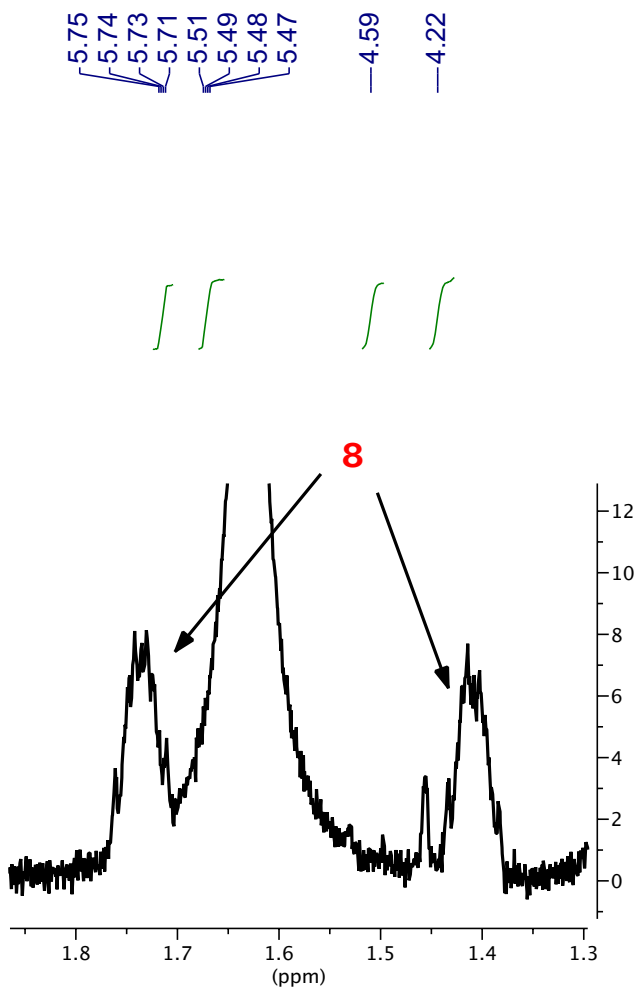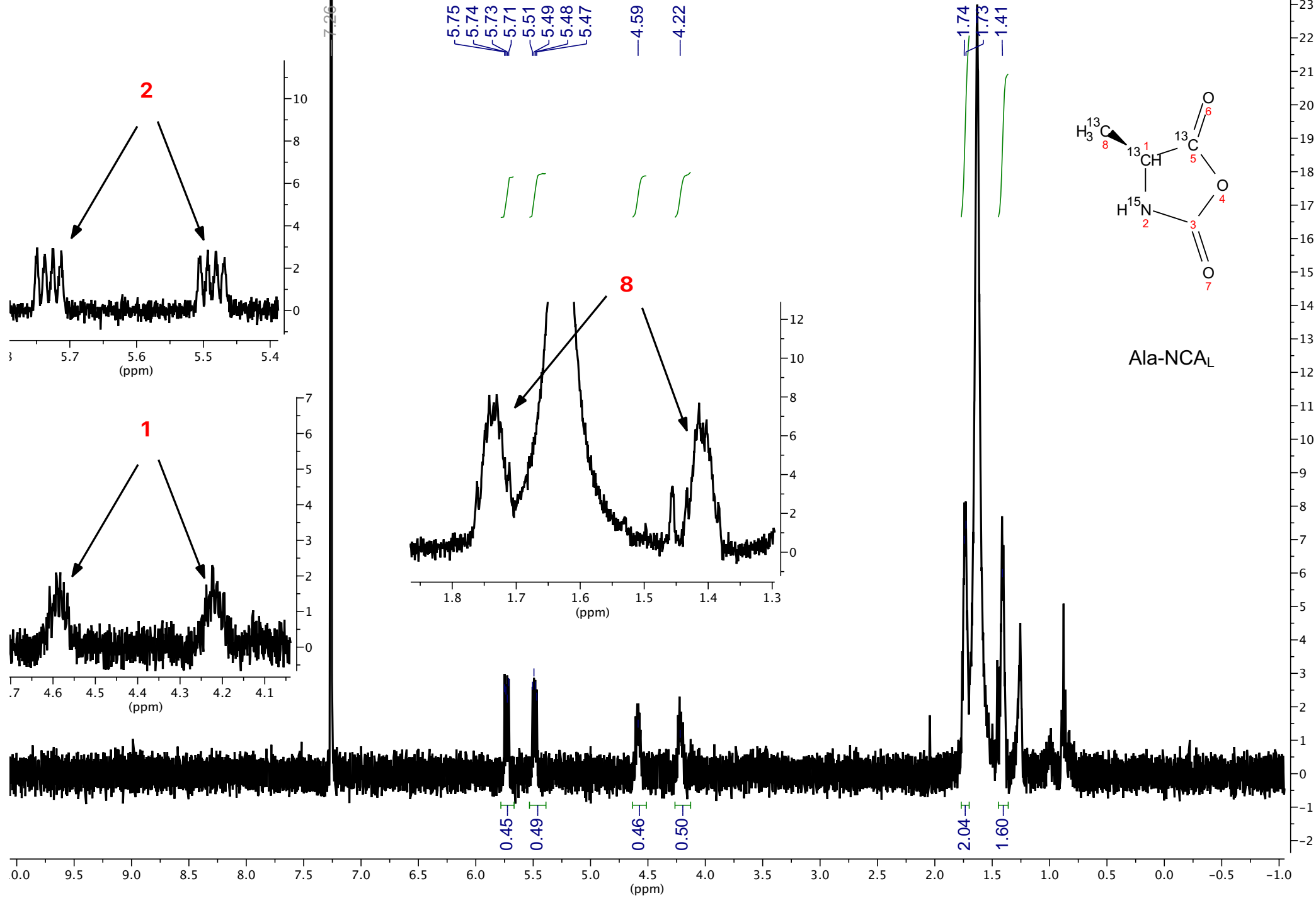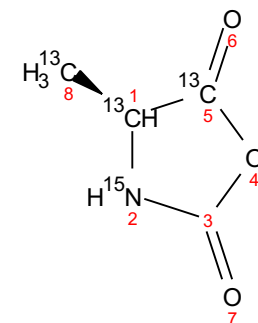

Ala-NCA<sub>L</sub>

Supplement: Supplementary file 3 — Supplementary Data 1 [file 42004_2025_1634_MOESM3_ESM.pdf]
